# Mathematical Modeling of Tumor Growth in Preclinical Mouse Models with Applications in Biomarker Discovery and Drug Mechanism Studies

**DOI:** 10.1158/2767-9764.CRC-24-0059

**Published:** 2024-08-29

**Authors:** Huajun Zhou, Binchen Mao, Sheng Guo

**Affiliations:** Crown Bioscience Inc., Suzhou, China; Crown Bioscience Inc., Suzhou, China; Crown Bioscience Inc., Suzhou, China

## Abstract

Oncology drug efficacy is evaluated in mouse models by continuously monitoring tumor volumes, which can be mathematically described by growth kinetic models. Although past studies have investigated various growth models, their reliance on small datasets raises concerns about whether their findings are truly representative of tumor growth in diverse mouse models under different vehicle or drug treatments. In this study, we systematically evaluated six parametric models (exponential, exponential quadratic, monomolecular, logistic, Gompertz, and von Bertalanffy) and the semiparametric generalized additive model (GAM) on fitting tumor volume data from more than 30,000 mice in 930 experiments conducted in patient-derived xenografts, cell line–derived xenografts, and syngeneic models. We found that the exponential quadratic model is the best parametric model and can adequately model 87% studies, higher than other models including von Bertalanffy (82%) and Gompertz (80%) models; the latter is often considered the standard growth model. At the mouse group level, 7.5% of growth data could not be fit by any parametric model and were fitted by GAM. We show that endpoint gain integrated in time, a GAM-derived efficacy metric, is equivalent to exponential growth rate, a metric we previously proposed and conveniently calculated by simple algebra. Using five studies on paclitaxel, anti-PD1 antibody, cetuximab, irinotecan, and sorafenib, we showed that exponential and exponential quadratic models achieve similar performance in uncovering drug mechanism and biomarkers. We also compared exponential growth rate–based association analysis and exponential modeling approach in biomarker discovery and found that they complement each other. Modeling methods herein are implemented in an open-source R package freely available at https://github.com/hjzhou988/TuGroMix.

**Significance:**

We present a general strategy for mathematically modeling tumor growth in mouse models using data from 30,000 mice and show that modeling and nonmodeling approaches are complementary in biomarker discovery and drug mechanism studies.

## Introduction

The success of oncology drug development hinges on the proper use of preclinical models, including *in vitro* cell line and organoid models, as well as *in vivo* xenograft and allograft mouse models (1–4). Resembling primary tumors more faithfully in histopathology and genomics, mouse models serve as a better tool for translational oncology by exhibiting more clinically similar drug responses (5, 6). For *in vivo* pharmacology studies, tumor-bearing mice are treated with drugs, and efficacy is monitored by continuingly measuring tumor volumes over weeks or even months. This results in mouse-specific longitudinal tumor growth data, which can be mathematically modeled by kinetic equations for better efficacy evaluation and biomarker discovery compared with simple readouts such as tumor growth inhibition. For example, we have previously shown that using linear mixed models, *EGFR* gene expression was identified as the best single-gene biomarker for predicting the efficacy of Erbitux, an EGFR mAb, on patient-derived xenograft (PDX) models of gastric cancer. In contrast, in naïve association analysis using tumor growth inhibition, there were more than 100 genes with falsely better statistical correlation significance than *EGFR* (6).

There are two main categories of mathematical modeling for tumor growth. In the parametric approach, tumor volume *V* is a function of time *t*, and the instantaneous growth rate $\frac{dV}{dt}$ is described by a differential equation from which tumor volume at any time can be derived as a parametric kinetic equation (7). The exponential model is the simplest kinetic equation, and its instantaneous growth rate is proportional to the tumor volume. In theory, this means the tumor can grow with acceleration infinitely; however, in the real world, tumor growth slows down in the later observational period before mouse euthanasia due to large tumor volume. In the logistic and Gompertz models, tumor growth is constrained by a maximal volume called the carrying capacity and follows a sigmoid growth curve with an inflection point. The monomolecular model is also constrained by a maximal volume, but its growth curve is a concave function without an inflection point. The von Bertalanffy model has one more parameter than the former three models, resulting in a curve shape that is intermediate between those of the former three models (8, 9).

Attempts to parametrically model tumor growth curves with a maximal volume constraint can be traced back to early 20th century when animal tumor growth was first modeled using the Gompertz model (10). Since then, the Gompertz model has been widely applied in modeling tumor growth curves [reviewed in ref. (11)]. Several studies have compared the Gompertz model with other models, such as the logistic and von Bertalanffy models, and found that these other models usually describe the data rather well, but the Gompertz model is still preferred (11–13). In recent systematic studies of parametric model comparison based on Akaike information criterion (AIC) and predictive power, the Gompertz model seemed to be one of the best models (7, 14, 15). However, these studies were mostly based on particular cancer types in small datasets. Furthermore, comprehensive comparisons between models using the confidence set for the Kullback–Leibler (K-L) best model and lack-of-fit test are, to our knowledge, lacking. A systematic study on large tumor growth datasets across a wide collection of mouse models under both vehicle and drug treatments is therefore needed to understand general tumor growth kinetics for suitable parametric modeling.

On the other hand, tumor growth is not assumed to follow any parametric form but is instead described using local nonlinear regression over time with kernel- or spline-based smoothing techniques. Subsequently, efficacy readouts can be defined, such as KuLGaP from the Gaussian process model (16) and endpoint gain integrated in time (eGaIT) from the generalized additive mixed model (ref. 17). Such nonparametric or semiparametric methods are flexible and can accommodate growth curves with irregular shapes, but they are more computationally intensive and require more data for good performance due to increased model complexity. They may also offer limited, if any, improvement on growth data that can be well modeled by parametric methods. Additionally, these methods can be cumbersome to interpret, especially when multiple covariates are involved, such as tumor feature and genomic data.

In the first part of this work, we compare six parametric models for their fitting on tumor growth data in PDXs, human cancer cell line–derived xenografts (CDX), and mouse cancer cell line–derived syngeneic mice, covering a variety of cancer origins, with more than 10,000 mice in each category. We show that the exponential quadratic model can fit most of the tumor growth data well. In the second part, we demonstrate that for irregular curves that cannot be adequately fit by any of the six parametric models, the generalized additive model (GAM) can be used effectively. In the last part, we use case studies to explore drug mechanisms of action (MoA) and biomarker discovery by using the exponential quadratic model, the exponential model, two efficacy metrics [eGaIT and exponential growth rate (eGR)] in association analysis and show that these methods are complementing each other.

## Materials and Methods

### Tumor volume data acquisition

Historical efficacy data were collected and anonymized for PDX, CDX, and syngeneic models to remove drug information so only treatment information as either “vehicle” or “drug” was kept. Drug efficacy was assessed every few days by tumor volume (*TV*) which was measured using a caliper to obtain the tumor length (*L*) and width (*W*), applying the formula TV = (*L* × *W*^2^)/2. A mouse was euthanized when the TV exceeded 2,000 to 3,000 mm^3^. For each model type, we randomly selected slightly more than 10,000 mice with initial tumor volumes in the range of 50 to 150 mm^3^ and with at least five TV data points. A group of mice receiving the same treatment in an experiment is defined as an *experimental group*. All animal experiments were conducted in the specific pathogen-free animal facility at Crown Bioscience, with approval from the Institutional Animal Care and Use Committee.

### Parametric model fitting of tumor volume data

#### Initial model fitting

We used six parametric models to fit tumor growth kinetics on tumor volumes by day (Table 1). We started by assuming a statistical model for an experimental group of mice:

$$V_{ij}=\;f\left(t_{j},\beta \right)+\epsilon_{ij}$$ (A)

in which $V_{ij}$ is the tumor volume for mouse *i* on day *j*, with an independently and normally distributed homoscedastic error term $\epsilon_{ij}$ ∼ (i.i.d) $N\left(0,\sigma^{2}\right)$; f(t,β) is one of the six parametric models. $f\left(t_{j},\beta \right)$ is the fitted tumor volume on day *j* for this group of mice. The maximum likelihood estimate for β is equivalent to ordinary least square estimate for β (18). Therefore, the objective function to be minimized is “residual sum of squares.”

**Table 1:** Six parametric models for fitting tumor growth data

Model	Differential equation	Parametric equation
Exponential	$\frac{dV\left(t\right)}{dt}=\alpha V\left(t\right)$	$V\left(t\right)=V_{0}e^{\alpha t}$
Gompertz	$\frac{dV\left(t\right)}{dt}=\alpha \log\left(\frac{K}{V\left(t\right)}\right)V\left(t\right)$	$V\left(t\right)=Ke^{-\log\left(\frac{K}{V_{0}}\right)*e^{-\alpha t}}$
Logistic	$\frac{dV\left(t\right)}{dt}=\alpha \left(1-\frac{V\left(t\right)}{K}\right)V\left(t\right)$	$V\left(t\right)=\;\frac{K}{1+\frac{K-V_{0}}{V_{0}}e^{-\alpha t}}$
Monomolecular	$\frac{dV\left(t\right)}{dt}=\alpha \left(K-V\left(t\right)\right)$	$V\left(t\right)=K-\left(K-V_{0}\right)e^{-\alpha t}$
von Bertalanffy	$\frac{dV\left(t\right)}{dt}=\eta {V\left(t\right)}^{m}-\kappa V\left(t\right)$	$V\left(t\right)={\left(\frac{\eta }{\kappa }-\left(\frac{\eta }{\kappa }-{V_{0}}^{1-m}\right)e^{-\left(1-m\right)\kappa t}\right)}^{\frac{1}{1-m}}$
Exponential quadratic	$\frac{dV\left(t\right)}{dt}=\alpha V\left(t\right)+\beta V\left(t\right)t$	$V\left(t\right)=V_{0}e^{\alpha t+\frac{1}{2}\beta t^{2}}$

The fitting was performed using the lm() function for exponential and exponential quadratic models and the optim() function for the other models in the *R* environment (version 4.1.0). We applied logarithm transformation of tumor volume first. In optim(), we used the default Nelder–Mead optimization method and set hessian = TRUE to calculate Fisher information. The maximum iteration was set to 10,000. Parameter scaling was used, as it was essential for finding a reliable solution when initial parameters were not optimized (19). The scaling is based on the scale of the parameters. For example, $\beta_{0}$ is the maximum tumor volume it can potentially grow. We set the scale to 1,000. $\beta_{2}$ is typically the exponential growth rate, which is typically around 0.1. Nelder–Mead and other optimization methods obtained similar solutions, as long as parameter scaling was used (Supplementary Fig. S1). The parameter scales for each parametric model are listed in Supplementary Table S1. Parameter initializations are summarized in Supplementary Table S2.

To further ensure that minimization of objective function was achieved, we conducted a grid search for each parameter initialization. For a grid of a single parameter, 21 (for the exponential model) or 11 (for other models) within ±10 scaled range of each initialized parameter were used. For $\beta_{3}$ in the von Bertalanffy model, it equals to parameter *m*, which determines the shape of the curve. *m* approaching the limit of 1 is the Gompertz model; *m* = 2 is logistic; *m* = 0 is monomolecular. Therefore, 0.5, 1.5, 2, and 4 were chosen for initialization. Furthermore, the optimized parameters must meet the conditions listed in Supplementary Table S2 (e.g., initial volume > 0 and maximum volume be positive), and best optimization was only picked from results that fell within these conditions. Except that the von Bertalanffy model has 18 cases of reaching the iteration limit, all other models converged for all 4,313 cases by the default relative convergence tolerance.

#### Quantifying predictability

The first three quarters of the observation period were used for modeling construction using the same method as *initial model fitting.* Then the final one fourth of the growth curve is predicted by the constructed model. Prediction accuracy is quantified either by the last time point deviation from the actual tumor volume or root mean square error (RMSE) of the second part tumor volumes in the log scale.

#### Variance estimation and coefficient of variation

Asymptotic variance of parameters was obtained by taking the diagonal of the inverted Hessian matrix from optim() and multiplied by 2 ${\hat{\sigma }}^{2}$, according to the log-likelihood function of residuals. ${\hat{\sigma }}^{2}$ was estimated by the residual sum of squares divided by N-p (in which *N* is the data size and *p* is the number of parameters). The coefficient of variation (CV) is calculated by taking the square root of variance and divided by parameter estimate.

#### Variance stabilization transformation and model fitting on log-transformed tumor volume data

Due to the violation of the constant variance assumption, we used variance stabilization transformation (20), assuming that the error is proportional to tumor volume to the order of γ:

$$V=\;f\left(t,\beta \right)+f{\left(t,\beta \right)}^{\gamma }\epsilon$$ (B)

$$\text{Var }\left(V\right)=f{\left(t,\beta \right)}^{2\gamma }\text{Var }\left(\epsilon \right)=E{\left(V\right)}^{2\gamma }\sigma^{2}=\mu^{2\gamma }\sigma^{2}$$ (C)

in which $E\left(V\right)=\mu$, $\text{Var }\left(\epsilon \right)=\sigma^{2}$. Assume transformation *g*(*V*) will result in constant variance that is unrelated to μ. By Taylor expansion, $g\left(V\right)\approx g\left(\mu \right)+g^{\prime }\left(\mu \right)\left(V-\mu \right)$; then E(g(V))≈g(μ), $\text{Var }\left(g\left(V\right)\right)\approx g^{\prime }{\left(\mu \right)}^{2}\text{Var }\left(V\right)=g^{\prime }{\left(\mu \right)}^{2}\mu^{2\gamma }\sigma^{2}=\text{constant}$. Subsequently, $g^{\prime }\left(\mu \right)=\frac{C}{\mu^{\gamma }},\;g\left(\mu \right)=\;\int \frac{C}{\mu^{\gamma }}d\mu$, in which C is a constant. γ=0.75, 1, and  1.25 correspond to transformation $g\left(V\right)=\;V^{\frac{1}{4}},\;\;\log V,\;\;\text{and}\;V^{-\frac{1}{4}}$, respectively. We transformed tumor volume and refit all experimental groups with all six parametric models using the same optimization method with grid search, except for log-transformed tumor volumes, for which linear regression [lm() function in the R package] was used for exponential and exponential quadratic models, as log transformation converts exponential and exponential quadratic models to linear models. The log transformation also involves reparameterization of models (Supplementary Table S3).

### Model selection

#### AIC and confidence set for the K-L best model

We used AIC for comparing and selecting parametric models for an experimental group. AIC connects the relative expected K-L distance in information theory with maximized log-likelihood by Akaike (21) and is defined as

AIC= −2∗log(L)+2K (D)

in which *L* is the likelihood of a fitted parametric model and *K* is the number of parameters in the model (including the variance parameter $\sigma^{2}$). For model estimated by maximum likelihood,

$$AIC=\;N\left(\log\left(2\pi \right)+1\right)+N\log\left(\frac{\sum_{i=1}^{N}{\left(y_{i}-f\left(t_{i},{\hat{\beta }}_{MLE}\right)\right)}^{2}}{N}\right)+2K$$ (E)

If response variable *y* is transformed to g(y), then AIC calculation is slightly modified because of the extra derivative term in the likelihood function (21):

$$AIC=\;N\left(\log\left(2\pi \right)+1\right)-2\sum_{i=1}^{N}\log\left(g^{\prime }\left(y_{i}\right)\right)+N\log\left(\frac{\sum_{i=1}^{N}{\left(g\left(y_{i}\right)-f\left(t,{\hat{\beta }}_{MLE}\right)\right)}^{2}}{N}\right)+2K$$ (F)

in which *N* is the total number of tumor volume data points in the experimental group. In addition to AIC, we also evaluated AICc (a second-order variant of AIC, suitable for small samples) and Bayesian information criterion (BIC):

$$AICc=AIC+\;\frac{2K\left(K+1\right)}{n-K-1}$$ (G)

BIC=-2 log(L) + K log(n) (H)

in which *n* is the number of data points for model fitting. Likelihood functions and other transformations of tumor volume *V* are listed in Supplementary Table S4.

However, the AIC-minimum (AIC-min) model is not necessarily the best model. If we define the best model as the model that has the minimal K-L distance to the true model that generates the observed data, then similar to the concept of “confidence interval” for point estimate, we can obtain a “confidence set” for the K-L best model (21). Among the candidate models, there is a K-L best model and an AIC-min model, and their AIC difference is $\Delta_{p}=AIC_{K-L\;best}-AIC_{\text{min}}$. Here, $\Delta_{p}$ is a statistic, and the 95% percentile of $\Delta_{p}$ can be used as a cutoff for including candidate models into the confidence set. The 95% percentile of $\Delta_{p}$ is generally less than 7 (21). As a rule of thumb, models in which AIC differences are less than 7 have some support and should be considered equally plausible models (22). We thus calculated the likelihood ratio between each model and the AIC-min model. The likelihood ratio is defined by

$$\frac{L\left(g_{i}|x\right)}{L\left(g_{\min}|x\right)}=\frac{\exp\left(\left(-1/2\right)\;\text{AI}C_{i}\right)}{\exp\left(\left(-1/2\right)\;\text{AI}C_{\min}\right)}=\exp\left(\;-\frac{1}{2}\left(\text{AI}C_{i}-\text{AI}C_{\text{min}}\right)\right)\equiv \exp\left(-\frac{1}{2}\text{ΔAI}C_{i}\right)$$ (I)

If the likelihood ratio is greater than 0.03 (corresponding to $\Delta AIC_{k}=7$), then the model is included in the confidence set for the K-L best model (21, 22). Confidence sets for all experimental groups were calculated.

#### Lack-of-fit F-test

The residual sum of squares equals to the sum of pure error sum of squares and lack-of-fit sum of squares (9):

$$\overset{m}{\underset{t=1}{\sum }}\overset{n_{t}}{\underset{u=1}{\sum }}{\left(V_{tu}-{\hat{V}}_{t}\right)}^{2}\equiv \overset{m}{\underset{t=1}{\sum }}\overset{n_{t}}{\underset{u=1}{\sum }}{\left(V_{tu}-{\bar{V}}_{t}\right)}^{2}+\overset{m}{\underset{t=1}{\sum }}n_{t}{\left({\hat{V}}_{t}-{\bar{V}}_{t}\right)}^{2}$$ (J)

in which *t* denotes time points and *m* is the total number of time points. At each time point, there are $n_{t}$ number of measurements. ${\hat{V}}_{t}$ is the estimated tumor volume at time t by the statistical model, whereas ${\bar{V}}_{t}$ is the mean tumor volume at time *t*. Then the ratio *F*

$$F=\frac{\text{Lack‐of‐fit sum of squares}/(m-p)\;}{\;\;\text{Pure error sum of squares}/(N-m)}$$ (K)

follows an *F*-distribution with (m-p) and (N-m) degrees of freedom (*m* is the total number of time points, *N* is the total number of measurements, and *p* is the number of parameters in the statistical model or the effective degrees of freedom for the GAM). *P* value less than 0.05 is considered significant; i.e., the statistical model seems to be inadequate.

#### Projection of curves by Uniform Manifold Approximation and Projection

Each locally estimated scatterplot smoothing (LOESS) of irregular curves, defined as ones lacking of fit by any parametric models, was plotted in a 120 × 120 PNG image. The pixels of each image are flattened into a vector of variables, composing a row of the data matrix. After removing redundant columns of the data matrix, the resulting full-ranked matrix was used as an input for UMAP and principal component analysis to display the relative distance between the irregular curves (arXiv.1802.03426).

#### Logistic regression

Logistic regression of the following model was carried out using the glm() function in the R environment:

$$\log\left(\frac{p}{1-p}\right)=\beta_{0}+\beta_{1}num.\;mice+\beta_{2}obs.period+\beta_{3}max.\log\;TV+\beta_{4}s.d.\;\log\;TV+\beta_{5}mouse.model.type+\beta_{6}treatment$$ (L)

in which p is the probability of being predicted well, defined as the last time point prediction being within 25% of the actual mean tumor volume.

### Methods in biomarker discovery

#### Mixed-effects models

Given the longitudinal tumor growth data, repeated measurements of a single mouse are correlated, and thus, it is not appropriate to treat all measurements as independent observations. The linear mixed-effects model (LMM) is used to compare the tumor growth rate between treatment groups and screen for biomarkers (6). LMM can be used for both exponential and exponential quadratic terms of time. For biomarker screening analysis, the exponential model was specified as

$$\log\left(TV_{iemt}\right)=\beta_{0}+\beta_{1}\log\left(TV_{iem0}\right)+\beta_{2}Day_{t}+\beta_{3}Day_{t}*I\left(Treat_{i}\right)+\beta_{4}Day_{t}*Gene_{m}+\beta_{5}Day_{t}*I\left(Treat_{i}\right)*Gene_{m}+b_{0i|e,m}+b_{1i|e,m}Day_{t}+c_{0e|m}+c_{1e|m}Day_{t}+d_{0m}+d_{1m}Day_{t}$$ (M)

The exponential quadratic model is given as

$$\log\left(TV_{iemt}\right)=\beta_{0}+\beta_{1}\log\left(TV_{iem0}\right)+\beta_{2}Day_{t}+\beta_{3}Day_{t}*I\left(Treat_{i}\right)+\beta_{4}Day_{t}*Gene_{m}+\beta_{5}Day_{t}*I\left(Treat_{i}\right)*Gene_{m}+\beta_{6}Day_{t}^{2}+b_{0i|e,m}+b_{1i|e,m}Day_{t}+c_{0e|m}+c_{1e|m}Day_{t}+d_{0m}+d_{1m}Day_{t}$$ (N)

in which $TV_{iemt}$ is the tumor volume of mouse *i* in experiment *e* within model *m* on day *t*; $TV_{iem0}$ is the tumor volume on day 0; $Day_{t}$ is day variable on day *t*; $I\left(Treat_{i}\right)$ is the binary variable indicating whether the mouse *i* is drug-treated; $Gene_{m}$ is the specific gene expression in PDX model *m*; $b_{0i|e,m}$ is the random intercept for an individual mouse within experiment *e* and PDX model *m*; $c_{0e|m}$ is the random intercept for each experiment within PDX model *m*; $d_{0m}$ is the random intercept for PDX model *m*; and $b_{1i|e,m},\;c_{1i|e,m},\;{\text{and}\;d}_{1m}$ are random slopes for an individual mouse, experiment, and PDX model, respectively. *P*-value of $\beta_{5}$ is used for ranking genes.

#### GAM

The gam() function in the mgcv package in R was used for fitting data with GAM. The model is specified as

$$\log\left(TV_{it}\right)=\beta_{0}+f\left(Day_{t}\right)+\epsilon_{it}$$ (O)

For the tumor volume of mouse *i* on day *t*, $f\left(Day_{t}\right)$ is a smooth function of day. By software default, we used the thin-plate regression spline smooth function. The number of days in the experimental group was set as the dimension of its basis function space. AIC calculation was done by using the logLik.gam() function based on effective degrees of freedom that is corrected by a method developed by Wood and colleagues (23) from the mgcv package, and for log-transformed response variable, an amendment term was also added (see Eq. F).

#### eGR and eGaIT

eGR is an “average” growth rate, calculated from the area under the tumor growth curves (6, 24). Briefly, after connecting a tumor’s longitudinal log-transformed volume measurements by straight lines, the area under the log-transformed growth curve is calculated by summing up the areas of trapezoids and then subtracted by the rectangular area underneath (with days of the observation period as length and initial log tumor volume as height). Then it is divided by quadratic of days from the observation period. It approximates the growth rate of an exponentially growing tumor (growth rate defined as the α constant in the exponential term) with the same AUC. eGR for a tumor is defined as

$$\text{eGR}=\;\frac{\sum_{m=1}^{M}({\log V}_{m-1}+\log V_{m})(t_{m}-t_{m-1})/2-\log V_{0}\;\left(t_{m}-t_{m-1}\right)}{\left(1/2\right)\;t_{M}^{2}}$$ (P)

in which *m* represents each measurement, *M* represents the endpoint measurement, *V* represents the tumor volume, and *t* represents the observation time. The eGR ratio takes the median of all possible ratio combinations of eGR in the drug group over eGR in the vehicle group. The eGR difference takes the median of all possible combinations of eGR in the drug group subtracted by eGR in the vehicle group.

By Forrest and colleagues (17), eGaIT for experimental group *i* is defined as

$$\text{eGaI}T_{i}=\frac{1}{\left(1/2\right)\;t_{M}^{2}}\int_{0}^{t_{M}}g_{i}\left(t\right)-g_{i}\left(0\right)dt$$ (Q)

in which *g*_*i*_*(t)* is a GAM-fitted smooth function of log-transformed tumor growth curves in group *i*. We calculated the statistic using the maeve package developed by the authors (17).

#### Gene set enrichment analysis

Gene set enrichment analysis (GSEA; ref. 25) was carried out using the clusterProfiler R package (26) by inputting the gene expression value for about 12,000 genes sorted by their Spearman correlation with the eGR ratio/difference or eGaIT ratio or *t* value of the three-way interaction term Treatment:Gene:Day in LMM.

### Data availability

The parametric modeling methods are implemented in an R package called TuGroMix and is available at https://github.com/hjzhou988/TuGroMix. It also comes with some accessory functions to facilitate data transformation. Example real tumor growth and gene expression data were also provided to give users a demonstration on how to perform growth modeling and biomarker screening.

## Results

### Overview of tumor growth data

We collected tumor growth data (i.e., tumor volume in mm^3^) for three types of mouse tumor models: PDX, CDX, and syngeneic models, each with more than 10,000 mice covering multiple cancer origins (Table 2; Supplementary Fig. S2). We observed that PDX tumors usually grow more slowly than CDX tumors, whereas syngeneic tumors generally grow much faster than both the PDX and CDX tumors. Consequently, syngeneic mice were usually sacrificed in 2 weeks, compared with 3 weeks for CDX mice and 4 weeks for PDX mice. Final tumor volumes in syngeneic models are generally larger than in CDX models, and CDX tumor volumes are larger than PDX tumor volumes, even though the initial tumor volumes are in the reversed order. Notably, we only selected tumors with initial tumor volumes in the range of 50 to 150 mm^3^.

**Table 2:** Descriptive statistics of the mouse tumor model dataset

A. Number of models and mice by each mouse model type and treatment category								
	CDX		PDX		Syngeneic		Overall	
	Drug	Vehicle	Drug	Vehicle	Drug	Vehicle	Drug	Vehicle
Number of models	62	59	224	191	21	20	307	270
Number of mice	8,672	1,812	8,569	2,002	8,910	1,847	26,151	5,661

B. Summary statistics of experimental groups by mouse model type and treatment category						
	CDX		PDX		Syngeneic	
	Drug	Vehicle	Drug	Vehicle	Drug	Vehicle
n	1,031	226	1,328	336	1,147	245
Number of mice [mean (SD)]	8.4 (1.9)	8.0 (1.9)	6.5 (2.7)	6.0 (2.7)	7.8 (2.6)	7.5 (2.7)

C. Summary statistics of tumor volume measurements at the experimental group level by mouse model type and treatment category						
	CDX		PDX		Syngeneic	
	Drug	Vehicle	Drug	Vehicle	Drug	Vehicle
n	8,672	1,812	8,569	2,002	8,910	1,847
Number of measurements [mean (SD)]	8.1 (2.4)	7.6 (2.2)	10.5 (5.3)	8.6 (3.1)	7.5 (3.2)	6.7 (1.8)
Initial TV [mean (SD)]	149 (42)	151 (41)	155 (51)	154 (48)	94 (29.39)	95 (28)
Final TV [mean (SD)]	1,133 (881)	1,700 (868)	893 (823)	1,271 (832)	1,689 (1,090)	2,190 (979)
Day span [mean (SD)]	22.5 (7.8)	21.1 (7.8)	33.0 (18.5)	26.4 (11.2)	18.8 (11.0)	16.1 (6.8)

D. Cancer origins covered in each mouse model type			
	CDX	PDX	Syngeneic
Number of cancer origins	15	19	12

### Fitting tumor growth data by parametric models

We selected six parametric models for modeling tumor growth curves (Table 1). In addition to five traditional parametric models used in tumor modeling, we introduce the “exponential quadratic” model. In a log-transformed exponential model of tumor volume, which turns out to be a linear model, we add a quadratic term of time to account for the downward or upward curvature in the ending stage, a common practice in statistical modeling. This model has the advantage of being fit within the framework of linear regression, which can be solved analytically. The quadratic term, when traced back to the differential equation, represents the growth rate decrease proportional to time. Notably, unlike the other five parametric models, which do not contain the time term on the right side of differential equations, the exponential quadratic model is not based on an autonomous differential equation (11). Details on fitting these six parametric models to tumor growth data are described in “Materials and Methods” section.

We found that fitting results are sensitive to different fitting algorithms (Supplementary Fig. S1). However, parameter scaling minimizes these differences. We chose the Nelder–Mead algorithm for its robustness and efficiency. Even though we used the best guess for parameter initialization for each parametric model (Supplementary Table S2), we still performed a grid search for initial parameters surrounding the best guesses to obtain the best fitting result.

To assess the quality of model fitting, we manually examined a plot of residuals against fitted values and a normal Q–Q plot (representative plots in Supplementary Figs. S3 and S4). For the majority of model fittings, residuals exhibit a funnel-shaped structure, and normal Q–Q plots show deviation from the normal distribution, both indicating a violation of the constant variance assumption of the parametric models. We almost always observe a larger variance of tumor volumes at later time points.

To address this issue, we applied variance stabilization transformation on tumor volume data (20). To find an appropriate transformation, we assessed three values of the order term γ: 0.75, 1, and 1.25, corresponding to transformations of tumor volume to $V^{\frac{1}{4}},\;\log V,\;{\text{and}\;V}^{-\frac{1}{4}}$, respectively (Eqs. B and C). We note that when γ=1, we simply perform the log transformation of tumor volume. We used a K-L distance–based method to compare model fittings under the three γ values and generated a K-L best model confidence set for each experimental group. AIC values of all three transformations for a single parametric model were compared. As long as the model ΔAIC (difference between AIC and the AIC-min among the three transformations) was no more than 7, the model was included in the confidence set of K-L best model (see “Materials and Methods”).

**Figure 1:**
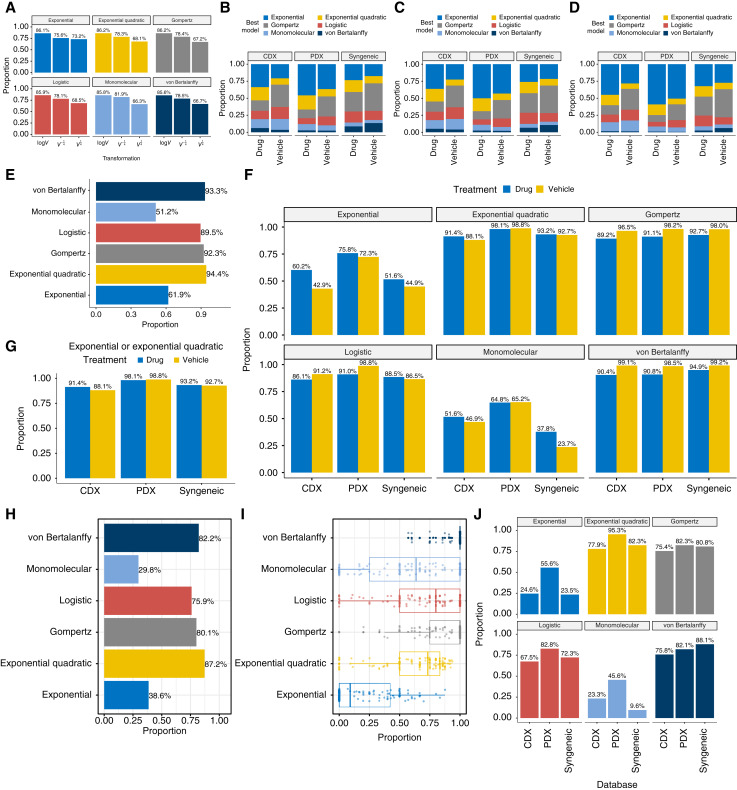
Parametric modeling of tumor growth data in mouse models. **A,** Proportion of each variable stabilization transformation $V^{\frac{1}{4}},\;\log V,\;\text{and}\;V^{-\frac{1}{4}}$ in the confidence sets of the K-L best model, for each parametric model. Log transformation accounts for the highest proportion (∼86%) in confidence sets for all six mathematical models. **B–D,** Proportion of each parametric model being the best model in terms of minimum AIC (**B**), AICc (**C**), and BIC (**D**), separated by mouse model type and treatment. **E,** Overall proportion of confidence sets that contain each of the six parametric models. **F,** Proportion of confidence sets that contain each of the six parametric models, separated by mouse model type and treatment. **G,** Proportion of confidence sets that contain either the exponential or exponential quadratic model. **H,** Proportion of experiments in which all experimental groups’ confidence sets contain each parametric model. **I,** For the 119 experiments in which not all experimental groups’ confidence sets contain the exponential quadratic model, the proportion of confidence sets containing each parametric model in each experiment. **J,** Proportion of experiments in which all experimental groups’ confidence sets contain each parametric model, separated by mouse model types.

We found that log transformation was present in about 86% of all confidence sets for all six mathematical models, higher than $V^{-\frac{1}{4}}$ (about 78%) and $V^{\frac{1}{4}}$ (about 68%; Fig. 1A). Although we could choose other γ values between 0.75 and 1.25, which might achieve better goodness of fit (in terms of AIC), for ease of implementation, we used γ=1, which corresponds to the log transformation.

We compared AIC difference (ΔAIC) between model fitting with and without log transformation of tumor volume and discovered that after log transformation, the AIC of 90% to 92% of model fits decreased (Supplementary Table S5). The median AIC reduction is around 33 (Supplementary Table S6), corresponding to a likelihood increase of about $1.47\times 10^{7}$ folds. The residual plots show increased homoscedasticity of variance (Supplementary Fig. S5), and the Q–Q plots suggest a normal distribution of residuals (Supplementary Fig. S6), indicating that log transformation of tumor volume data is necessary.

### Exponential quadratic model is the overall best parametric model for mouse studies

We used AIC, AICc, and BIC for model selection among the six parametric models. A model with the lowest AIC (AICc or BIC) model was selected as the best model. Overall, exponential, exponential quadratic, Gompertz, and logistic models accounted for the majority of best models (Fig. 1B–D). The AIC, AICc, and BIC results are similar. The proportion of exponential and exponential quadratic models being the best model is higher in the AICc and BIC results than in AIC. Exponential or exponential quadratic models are generally less favorable in vehicle groups than in drug groups, suggesting that tumor growth tends to get constrained by tumor environmental limits more in vehicle groups in which tumors grow faster.

More practically, if we loosen the criteria of selecting the best model by AIC-min and instead consider the confidence set for the K-L best model (i.e., all parametric models in the confidence set are equally good), then among the six parametric models, exponential quadratic, von Bertalanffy, Gompertz, and logistic models are contained in about 90% or more of the *experimental groups* (Fig. 1E). These four models are also vastly better than the other two parametric models (exponential and monomolecular models) when mouse model types and treatments were considered separately (Fig. 1F). The proportion of confidence sets that contain the exponential or exponential quadratic model is identical to the proportion of confidence sets that contain the exponential quadratic model only (Figs. 1E–G), indicating that exponential quadratic models cover all confidence sets that contain exponential models.

An experiment always has at least two experimental groups: one vehicle group and one drug group. There are frequently more than one drug groups, treated with either different drugs or the same drug with different dosages. We attempted to examine whether in an experiment, all groups can be fit by a single parametric model. In about 87% of all experiments (811 of 930), all groups’ confidence sets contain the exponential quadratic model (Fig. 1H). This is higher than those of von Bertalanffy (82%), Gompertz (80%), logistic (76%), and monomolecular (30%) models. For the remaining 119 experiments, the most “universal” model is von Bertalanffy, followed by Gompertz, logistic, and exponential quadratic (Fig. 1I). However, there are 20 experiments in which none of the six parametric models is present in all confidence sets.

In terms of mouse model type, the most universal model for CDX and PDX is the exponential quadratic model, as 78% and 95% of the experiments have the exponential quadratic model contained in all confidence sets, whereas von Bertalanffy is the most universal model for syngeneic models (Fig. 1J).

**Figure 2:**
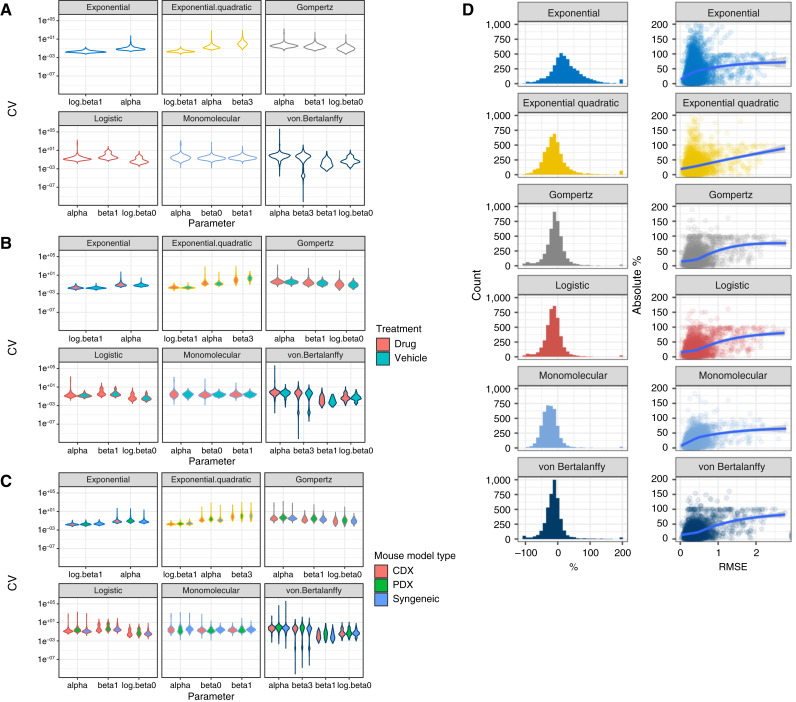
CV of the parameters and prediction accuracy for each parametric model. **A–C,** CV of each parameter in each parametric model, separated by treatment (**B**) and mouse model type (**C**). **D,** Left, Histograms of last time point prediction percentage deviation from the mean tumor volume (original scale; percentages greater than 200 are all constrained at 200). Right, RMSE of first three-fourth log tumor volume vs. last time point prediction percentage deviation from the mean tumor volume (original scale). Blue, LOESS regression lines.

To assess the reliability and identifiability of the models, we calculated the SE and the CV of the estimated parameters (Table 3). Higher SEs and CV imply less reliability and identifiability of the models and result in less precise predictions. We found that the linear regression–based exponential model has the smallest CV for the growth rate term α (Fig. 2A–C; Table 3). Logistic and exponential quadratic models have smaller CV than Gompertz and monomolecular models. von Bertalanffy has the largest CV for α and *β*_3_ overall. Treatmentwise, drug groups have larger CVs than vehicle groups (Fig. 2B). With regard to mouse model type, there is no consistent CV outperformance for any particular data type (Fig. 2C).

**Table 3:** Medians of estimated parameters of each mathematical model

Model	Parameter	Median estimate	Median SE	Median CV
Exponential	Log V_0_	5.04	0.0902	0.0181
	α	0.0820	0.00730	0.0698
Exponential quadratic	Log V_0_	4.93	0.106	0.0219
	α	0.127	0.0241	0.155
	*β*_3_	−0.00172	0.00110	−0.298
Logistic	Log K	7.64	0.321	0.0442
	*β*_1_	15.5	3.98	0.398
	α	0.149	0.0249	0.137
Gompertz	Log K	8.58	0.785	0.0893
	*β*_1_	3.84	0.745	0.224
	α	0.0414	0.0205	0.347
Monomolecular	K	1.29e5	1.39e3	0.196
	*β*_1_	1.28e5	1.43e3	0.219
	α	0.00132	0.00141	0.257
von Bertalanffy	Log β_0_	7.80	0.318	0.0452
	*β*_1_	−2.95	11.3	1.29
	α	0.0650	0.0665	0.481
	m	1.51	0.527	0.300

In conclusion, the exponential quadratic model is the overall best parametric model for mouse studies due to its performance on different mouse models, simplicity and interpretability, reliability, and identifiability.

### Gompertz and von Bertalanffy are best for their predictive capability

With regard to predictive capability of the six mathematic models, we used the first three fourths of the observational period to construct models and predicted tumor volumes for final one fourth. Prediction accuracy was shown as the percentage change at the last time point prediction (Fig. 2D) and RMSE of log-transformed tumor volume (Supplementary Fig. S7). We found that the exponential model usually overestimates tumor volume, whereas the other models tend to underestimate tumor volume [Fig. 2D (left)]. Gompertz and von Bertalanffy models have the best predictions, as their histograms are narrower and their modes are closest to zero.

To see whether there is a correlation between fitting the first three fourths of the data and prediction accuracy for the final one fourth, we plotted the RMSE of the first three fourths of the data versus the prediction percentage error and observed a positive correlation for all six models [Fig. 2D (right)]. Nevertheless, Gompertz, von Bertalanffy, and logistic models have lower prediction errors than the other three models when RMSE is less than 0.5 [see LOESS regression lines in Fig. 2D (right)], indicating better prediction accuracy for these three models. From the overall prediction RMSE of the final quarter of the data, we see similar performance, as Gompertz, von Bertalanffy, and logistic models have the lowest prediction RMSE (Supplementary Fig. S7A).

With regard to treatment, all mathematical models predicted less accurately for drug groups than for vehicle groups (Supplementary Fig. S7B). In terms of mouse model type, CDX and PDX models are generally more predictable than syngeneic models (Supplementary Fig. S7C).

We further examined factors associated with predictability. To see how the shape of the growth curve is related to prediction accuracy, LOESS regression lines of all log-transformed curves were stretched to fit 120 × 120 pixel images and were visualized in a UMAP projection space (Supplementary Fig. S8A). We defined “good prediction” as an absolute prediction error of the last time point of less than 25% for Gompertz, von Bertalanffy, logistic, and exponential quadratic models simultaneously. These models are mostly straight or bending down at a later stage (yellow lines in Supplementary Fig. S8A).

We conducted logistic regression to test what other factors are related to prediction and found that mouse model type, drug treatment, observation period length, and variance of log-transformed tumor volume data are all significantly related to prediction accuracy (Supplementary Fig. S8B). CDX and syngeneic models are less predictable than PDX models, and drug treatment is less predictable than vehicle control. These findings are consistent with the RMSE results (Supplementary Fig. S7).

### Growth data that are not well fitted by any parametric models

**Figure 3:**
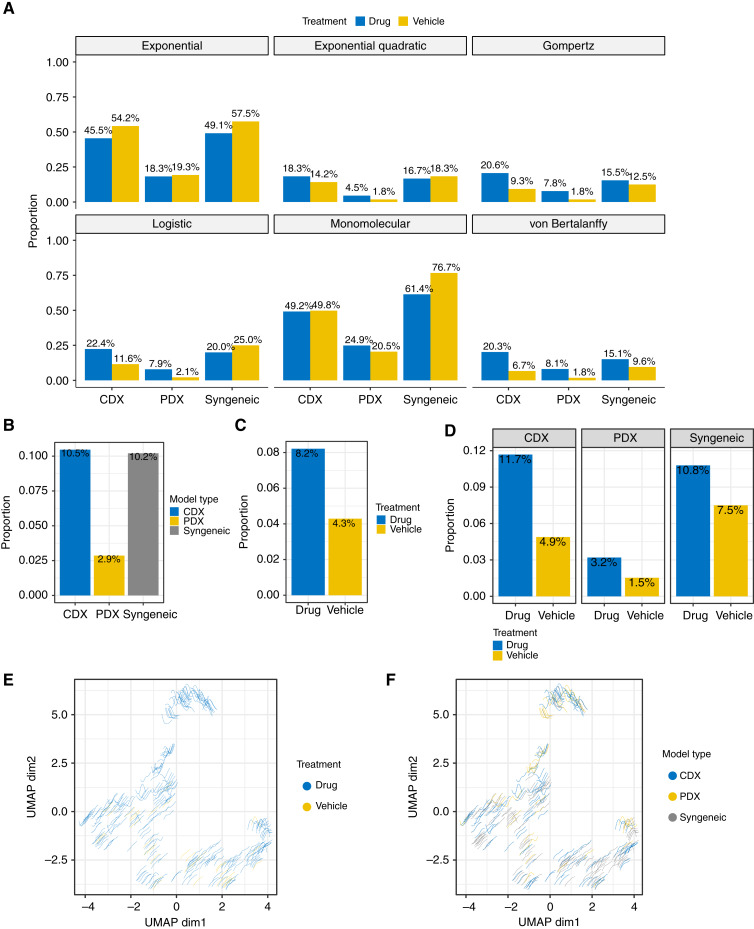
Lack-of-fit F-test. Proportion of group tumor volume data that lack fit by any of the six parametric models by lack-of-fit test, separated by **A** parametric model, mouse model type, and treatment; (**B**) mouse model type; (**C**) treatment by vehicle or drug; (**D**) mouse model type and treatments. **E** and **F,** UMAP of the irregular curves, colored by treatment (**E**) and model type (**F**).

To examine whether the six parametric models can adequately cover and fit all tumor curves, we used a lack-of-fit F-test, which tests whether a parametric model is adequate for a given set of tumor volume data [Draper and Smith (9)]. The exponential quadratic model seems the most “universal” model, with only 12.2% of growth data lacking fit, followed by von Bertalanffy (12.4%), Gompertz (12.8%), logistic (15.3%), exponential (37.3%), and monomolecular models (44.4%; Supplementary Table S7). Specifically, for PDX vehicle groups, exponential quadratic, von Bertalanffy, and Gompertz models have equally the lowest unfit rate (1.8%). However, for PDX drug groups, the exponential quadratic model has the lowest unfit rate (4.5%), compared with 7.8% for Gompertz and 8.1% for von Bertalanffy (Fig. 3A). von Bertalanffy and Gompertz models fit syngeneic data better than the exponential quadratic model for both vehicle and drug groups (Fig. 3A). For CDX data, von Bertalanffy and Gompertz fit vehicle groups better than the exponential quadratic model but not so for CDX drug groups (Fig. 3A). These model performance results are consistent with the K-L best model confidence set results (Fig. 1E and F).

With regard to tumor origin, the most unfit mathematical models are still exponential and monomolecular models across different tumor origins (Supplementary Figs. S9 and S10). The differences between the other four mathematical models are not large across different tumor origins (Supplementary Figs. S9 and S10). For vehicle groups, only a small proportion of tumors in lymphoma, gastric cancer, and colorectal cancer in PDX models lack fit from the other four models [Supplementary Fig. S9 (PDX)]. However, for drug groups, various cancer origins lack fit from the other four models [Supplementary Fig. S10 (PDX)]. Similar results were observed for CDX (CDX panels in Supplementary Figs. S9 and S10).

There are 315 groups (7.5% of the data) that lack fit by any of the six parametric models. Specifically, approximately 10% of the data in CDX and syngeneic models lack fit by any of the six parametric models, which is much higher than in PDX models (3%; Fig. 3B). Drug groups have more irregular tumor growth curves than vehicle groups (Fig. 3C and D), suggesting that tumor growth kinetics under drug treatment are more difficult to model. The irregular log-transformed curves can be visually categorized into two major groups by UMAP (Fig. 3E) or principal component analysis (Fig. S11) projections: wavy and oscillating curves (Fig. 3E; UMAP dim2 >1), and growing curves that are “S”-shaped or reverse “S”-shaped (Fig. 3E; UMAP dim1 <2 and dim2 <0). Almost all of the first type of curves belong to drug treatment groups (Fig. 3E), consistent with our observation that drug effects on tumor growth are more difficult to model (Fig. 3C and D). The syngeneic and CDX curves account for the majority of the second type of curves (Fig. 3F), and many of them are vehicle groups, suggesting that cell line–based tumors grow more differently from primary tumors and harder to model. In the drug groups, the reversed S-shaped curves could be due to slow-acting drug effects.

We selected these curves from UMAP (Supplementary Fig. S11). Using a method developed by Harris and colleagues (27), we attempted to determine the start time for drug effects. Among the selected drug group curves, about 64% of them have a start time less than one third of the full treatment period, suggesting that we could potentially discard the initial treatment period for those curves and fit the exponential or exponential quadratic model for the remaining part of those curves. The poor fitting can also be due to some mice surviving much longer than the others [Supplementary Fig. S12 (upper)], with about 12 such cases. The rest of the fittings are mostly homogeneous between mice [Supplementary Fig. S12 (lower)], preventing independent modeling with two or more mathematical models for these curves. The common characteristic of these curves is that the bending changes from concave to convex (and some back to concave again). This is likely due to delayed or short drug effects but can also be due to some intrinsic nature of the tumor, as we see this in the vehicle group as well (Supplementary Fig. S13). Notably, the log-transformed logistic, Gompertz, and monomolecular curves are all concaves (Supplementary Fig. S14). This may explain why these models cannot fit this type of growth curves well.

### GAM can be used to fit irregular growth data

**Figure 4:**
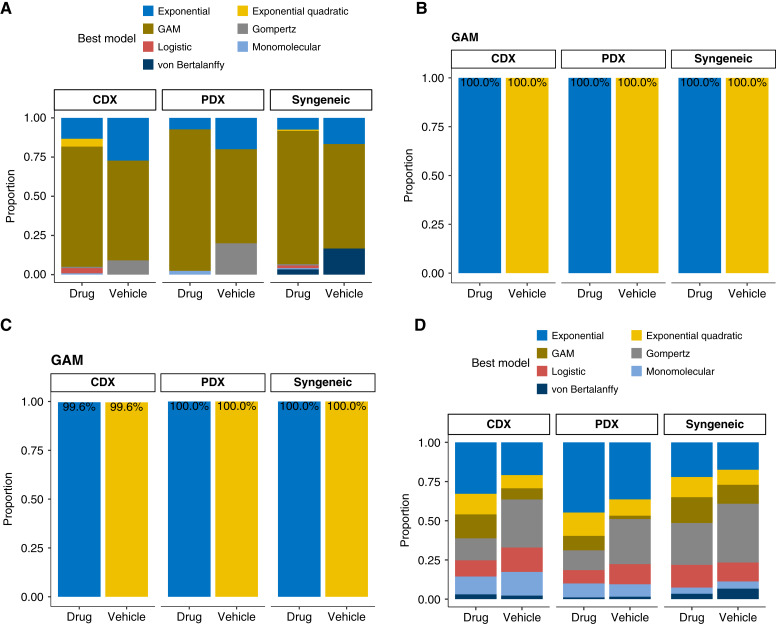
GAM modeling of tumor growth data in mouse models. **A,** AIC-min model proportion in the 7.5% experimental groups that lack fit by any of the six parametric models. **B,** Proportion of confidence sets that contain GAM in the 7.5% experimental groups that lack fit by any of the six parametric models. **C,** Proportion of confidence sets that contain GAM in datasets. **D,** AIC-min model proportion in all datasets.

For the 7.5% (315 experimental groups) of the growth data that lacked fit by any of the six parametric models, we used the GAM (see “Materials and Methods”). GAM is essentially a linear model, and when used here, the log-transformed tumor volume depends linearly on some smooth functions of day. In GAM, overfitting is reduced by the addition of penalization for wiggliness. After fitting those 315 experimental groups by GAM, only about 8% (25 experimental groups) still lacked fit. Therefore, GAM can fit most of the irregular curves. In fact, GAM’s AIC is the minimum AIC for the majority of the 315 experimental groups that lacked fit (Fig. 4A). In terms of the confidence set for the K-L best model, GAM is in all confidence sets for these 315 experimental groups, and almost all confidence sets in the context of the whole dataset (Fig. 4B and C), even though the proportion of GAM being minimum AIC considering the whole dataset is somewhat low (Fig. 4D). Therefore, GAM can be a universal model for tumor growth curve modeling, but it suffers from slowing running time and difficult parameter and model interpretation, making it less favorable whenever a good parametric model is available.

### Application of tumor growth curve models in biomarker discovery

#### Comparison between exponential and exponential quadratic models

**Figure 5:**
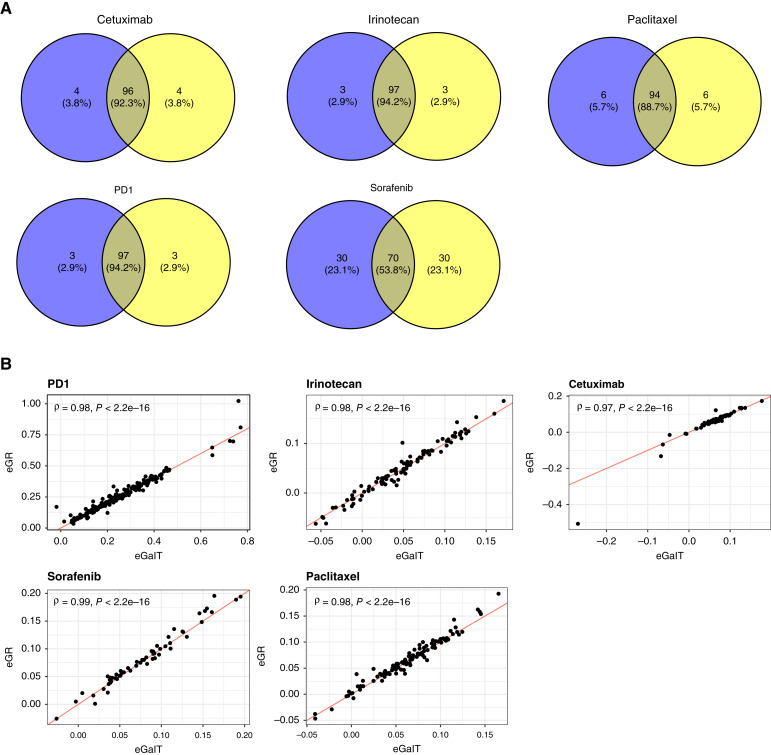
Application of tumor growth curve models in biomarker discovery. **A,** Venn diagrams of top 100 genes identified by exponential and exponential quadratic models for the five datasets. **B,** Scatter plots of eGaIT and eGR statistics for PD1, irinotecan, cetuximab, sorafenib, and paclitaxel datasets. ρ, Spearman correlation coefficient. The line indicating that eGR equals eGaIT is shown in red.

As the exponential quadratic model is generally the best model for PDX, we compared its performance with the exponential model in several biomarker discovery case studies. A mixed-effects model framework was used because of the longitudinal nature of measurement of each tumor (see “Materials and Methods”). The identified top genes are very similar between these two models (Fig. 5A), even though the exponential model fit far less well than the exponential quadratic model (Supplementary Table S8). The top identified pathways from GSEA (25) are highly identical between these two models (Supplementary Fig. S15A–S15E). These results indicate that the exponential model is adequate for identifying drug target–related biological pathways and biomarkers and is preferred in practice because of its simplicity for interpretation and shorter model fitting time.

#### eGaIT and eGR are two AUC-based growth curve statistics

In a study by Forrest and colleagues (17), they introduced a tumor growth rate summary statistic, eGaIT, based on the area under tumor growth curves. They fit growth curves using GAM and calculated the growth rate statistic based on the area under fitted smooth curves. This statistic is similar to our eGR statistic, except that we directly calculate the AUC by summing up the area of individual trapezoids (6, 24). The calculated statistic equals to the growth rate of a tumor growing exponentially (i.e., a straight line of log-transformed tumor volume) with the same AUC. They also showed that GAM-based eGaIT yields a greater t-statistic than the LMM-based slope, thus having greater power.

Here, we compared calculated eGaIT and eGR for the five datasets. The two statistics are highly correlated (Fig. 5B). Using the eGaIT or eGR ratio as a surrogate for drug efficacy, we calculated the Spearman correlation between each gene’s expression and drug efficacy and ordered genes according to the correlation coefficient. GSEA identified biological processes and pathways directly related to MoA of the drugs (Supplementary Fig. S16A–S16E). These results demonstrate that eGaIT and eGR are essentially equivalent both mathematically and numerically for biomarker discovery, and eGR is preferred because of its simply fast calculation.

#### Comparison of biomarker discovery between eGR and exponential model–based methods

To see if there is a difference in the biomarker discovery between the parametric model–based exponential model (LMM) and the simple eGR metric, we used the same five case study datasets. We compared whether they identify the target-related terms through GSEA. We also added a method based on the correlation between eGR difference and gene expression. The eGR difference is the difference between treatment and control groups and is thus a more stable statistic than the eGR ratio when the control group’s eGR is small.

Among the five case studies, both methods identified the underlying biological mechanisms (Supplementary Fig. S17). However, the LMM did not identify extracellular matrix and growth factor–related terms in the cetuximab study, and the eGR ratio–based method did not identify immune response–related terms in the anti-PD1 study (Supplementary Fig. S17A–S17E). In the anti-PD1 study, the eGR difference identified some immune-related terms in Gene Ontology biological processes, whereas the eGR ratio did not, suggesting that the eGR difference is more robust than the eGR ratio.

In conclusion, there is no single best method in biomarker discovery. It is best practice to use all models simultaneously so that the results can be compared and may complement each other.

## Discussion

Based on more than 30,000 mouse tumor growth datasets, we explored parametric and semiparametric modeling of tumor growth. Using the confidence set for the K-L best model, we showed that logarithm transformation of tumor volume generally works well for variance stabilization for tumor volumes. The exponential quadratic model is the most applicable parametric model, followed by von Bertalanffy, Gompertz, logistic, and monomolecular models. von Bertalanffy and Gompertz may fit better for syngeneic and CDX vehicle groups than the exponential quadratic model but worse for drug groups. The lack-of-fit test showed the same ordering of the best parametric models. However, about 7.5% experimental groups lack fit from any of the six parametric models. GAM can be used to fit these irregular tumor curves in most cases, and GAM is in almost all confidence sets considering the whole dataset. However, GAM may not be preferred in most cases because of its fitting slowness, interpretation difficulty, and the availability of parametric models with comparable performance.

The Gompertz model was originally developed by Benjamin Gompertz in 1825 for describing the distribution of human age for actuarial purposes. In the early 1900s, biologists started to use it as a growth curve for biological phenomena (28). The Gompertz model was used for tumor growth as early as 1934 (10). Recent studies and surveys found that the Gompertz model seems to outperform other models in general (7, 11, 14, 15). In our study, judged by the K-L best model confidence set and the lack-of-fit test, Gompertz, together with von Bertalanffy and exponential quadratic models, are the best models for syngeneic, CDX, and PDX vehicle groups, consistent with previous results. With regard to forecasting tumor growth, Gompertz and von Bertalanffy outperform other models. For biomarker discovery and MoA studies, the exponential quadratic model is preferred over Gompertz and von Bertalanffy because of its computational convenience and interpretability, and the exponential model seems adequate as it generates results largely overlapping with those from the exponential quadratic model.

In the field of pharmacokinetics, there are more sophisticated models that explicitly model tumor heterogeneity, tumor biology process, and therapeutic effect [reviewed in ref. (29)]. These models can be complicated and difficult to apply in preclinical animal studies due to data insufficiency, overparameterization, and inadequacy for describing the potentially intricate multiple phases of tumor growth.

Systematic studies have recently begun on statistics for mouse clinical trials, including publications using the structured covariance matrix of error (30), linear mixed-effects model (6, 31), or GAM (17), all taking into account the longitudinal and multilevel nature of mouse clinical trial data. For drug efficacy evaluation and biomarker discovery in mouse clinical trials, we can use exponential and exponential quadratic models within the framework of the linear mixed-effects model, and we showed both are sufficiently powerful and simple to uncover predictive biomarkers and drug mechanisms.

We compared the results of biomarker discovery through eGR ratio/difference correlation analysis and LMM and found that both methods identified largely overlapping target-related pathways and can be used simultaneously for overall better performance. There are still some differences that may be caused by several factors, including violation of the normality assumption in LMM, information loss in summary statistic, and outliers in datasets.

In summary, our extensive analysis provides a thorough evaluation of both parametric and semiparametric models, highlighting their strengths and limitations. Our findings underscore the importance of model selection tailored to specific experimental conditions and demonstrate the utility of integrating eGR-based metrics with parametric modeling for enhanced biomarker discovery and drug mechanism investigation. The implementation of these methods in the open-source R package TuGroMix ensures accessibility and encourages further innovation in the field.

## Supplementary Material

Supplementary Tables Supplementary Tables 1 to 8

Figure S1 Figure S1

Figure S2 Figure S2

Figure S3 Figure S3

Figure S4 Figure S4

Figure S5 Figure S5

Figure S6 Figure S6

Figure S7 Figure S7

Figure S8 Figure S8

Figure S9 Figure S9

Figure S10 Figure S10

Figure S11 Figure S11

Figure S12 Figure S12

Figure S13 Figure S13

Figure S14 Figure S14

Figure S15 Figure S15

Figure S16 Figure S16

Figure S17 Figure S17
