# Supplementary material for: Mathematical Modeling of Tumor Growth in Preclinical Mouse Models with Applications in Biomarker Discovery and Drug Mechanism Studies
Source: Cancer Res Commun. 2024 Aug 29;4(8):2267–81. doi: 10.1158/2767-9764.CRC-24-0059 (PMC11360417; doi:10.1158/2767-9764.CRC-24-0059)
Supplement: Supplementary Tables — 1 to 8 [file crc-24-0059_supplementary_tables_suppst.docx]

**Supplementary Table 1. Reparameterization of each parametric model, and their new parameter scales**

|  | Re-parameterization | Re-parameterized form | Parameter scales |
| --- | --- | --- | --- |
| Exponential | $\beta_{1}=V_{0}$  $\beta_{2}=\alpha$ | $V\left( t \right)=\beta_{1}e^{\beta_{2}t}$ | $\beta_{1}=100$  $\beta_{2}=0.1$ |
| Exponential square | $\beta_{1}=V_{0}$  $\beta_{2}=\alpha$  $\beta_{3}=\frac{1}{2}\beta$ | $V\left( t \right)=\beta_{1}e^{\beta_{2}t+\beta_{3}t^{2}}$ | $\beta_{1}=100$  $\beta_{2}=0.1$  $\beta_{3}=0.001$ |
| Gompertz | $\beta_{0}=K$  $\beta_{1}=log(\frac{K}{V_{0}})$  $\beta_{2}=\alpha$ | $V\left( t \right)=\beta_{0}e^{-\beta_{1}e^{-\beta_{2}t}}$ | $\beta_{0}=1000$  $\beta_{1}=10$  $\beta_{2}=0.1$ |
| Logistic | $\beta_{0}=K$  $\beta_{1}=\frac{{K-V}_{0}}{V_{0}}$  $\beta_{2}=\alpha$ | $V\left( t \right)= \frac{\beta_{0}}{1+\beta_{1}e^{-\beta_{2}t}}$ | $\beta_{0}=1000$  $\beta_{1}=10$  $\beta_{2}=0.1$ |
| Monomolecular | $\beta_{0}=K$  $\beta_{1}={K-V}_{0}$  $\beta_{2}=\alpha$ | $V\left( t \right)=\beta_{0}-\beta_{1}e^{-\beta_{2}t}$ | $\beta_{0}=1000$  $\beta_{1}=1000$  $\beta_{2}=0.1$ |
| Von Bertalanffy | $\beta_{0}=\left( \frac{\eta}{\kappa} \right)^{\frac{1}{1-m}}$  $\beta_{1}=1-\left( \frac{V_{0}}{A} \right)^{1-m}$  $\beta_{2}=\left( 1-m \right)\kappa$  $\beta_{3}=m$ | $V(t)={\beta_{0}\left( 1-\beta_{1}e^{-\beta_{2}t} \right)}^{\frac{1}{1-\beta_{3}}}$ | $\beta_{0}=1000$  $\beta_{1}=10$  $\beta_{2}=0.1$  $\beta_{3}=1$ |

**Supplementary Table 2. Parameter initialization for each parametric model**

|  | $\beta_{0}$ | $\beta_{1}$ | $\beta_{2}$ | $\beta_{3}$ | Conditions |
| --- | --- | --- | --- | --- | --- |
| Exponential |  | $0.5(V_{min}+V_{max})$ | $\frac{V_{max}-V_{min}}{{(t}_{V_{max}}-t_{V_{min}})\beta_{1}}$ |  | $\beta_{1}>0$ |
| Exponential square |  | $0.5(V_{min}+V_{max})$ | $\frac{V_{max}-V_{min}}{{(t}_{V_{max}}-t_{V_{min}})\beta_{1}}$ | $\left( \frac{V_{max}-V_{min}}{t_{V_{max}}-t_{V_{min}}}-\beta_{1}V_{max} \right)/(V_{max}t_{N})$ |  |
| Gompertz | $V_{max}$ | $log(\frac{\beta_{0}}{V_{0}})$ | $\frac{\left( V_{max}-V_{min} \right)\beta_{1}}{{(t}_{V_{max}}-t_{V_{min}})\beta_{0}}$ |  | $\beta_{0}>0$  $\beta_{1}>0$ |
| Logistic | $V_{max}$ | $\frac{{\beta_{0}-V}_{0}}{V_{0}}$ | $\frac{V_{t_{2}}-V_{t_{1}}}{{(t}_{2}-t_{1})\beta_{0}}$ |  | $\beta_{0}>0$  $\beta_{1}>0$ |
| Monomolecular | $V_{max}$ | $\beta_{0}-V_{min}$ | $\frac{V_{max}-V_{min}}{{(t}_{V_{max}}-t_{V_{min}})\beta_{1}}$ |  | $\beta_{0}>0$  $\beta_{1}>0$ |
| Von Bertalanffy | $V_{max}$ | $1-\left( \frac{V_{0}}{\beta_{0}} \right)^{\frac{1}{1-\beta_{3}}}$ | $\frac{V_{max}-V_{min}}{{(t}_{V_{max}}-t_{V_{min}})\beta_{1}}$ | 0.5, 1.5, 2, 4 | $\beta_{1}>0, \beta_{3}<1$  $\beta_{1}<0, \beta_{3}>1$ |

**Supplementary Table 3. Reparameterization of each parametric model with tumor volume log-transformed, and their new parameter scales**

|  | Re-parameterization | Re-parameterized form | Parameter scales |
| --- | --- | --- | --- |
| Exponential | $\beta_{1}=V_{0}$  $\beta_{2}=\alpha$ | $\log V\left( t \right)=\log\beta_{1}+\beta_{2}t$ | $\log\beta_{1}=1$  $\beta_{2}=0.1$ |
| Exponential square | $\beta_{1}=V_{0}$  $\beta_{2}=\alpha$  $\beta_{3}=\frac{1}{2}\beta$ | $\log V\left( t \right)=\log\beta_{1}+\beta_{2}t+\beta_{3}t^{2}$ | $\log\beta_{1}=1$  $\beta_{2}=0.1$  $\beta_{3}=0.001$ |
| Gompertz | $\beta_{0}=K$  $\beta_{1}=log(\frac{K}{V_{0}})$  $\beta_{2}=\alpha$ | $\log V\left( t \right)=\log\beta_{0}-\beta_{1}e^{-\beta_{2}t}$ | $\log\beta_{0}=1$  $\beta_{1}=10$  $\beta_{2}=0.1$ |
| Logistic | $\beta_{0}=K$  $\beta_{1}=\frac{{K-V}_{0}}{V_{0}}$  $\beta_{2}=\alpha$ | $\log V\left( t \right)=\log\beta_{0}-\log(1+\beta_{1}e^{-\beta_{2}t})$ | $\log\beta_{0}=1$  $\beta_{1}=10$  $\beta_{2}=0.1$ |
| Monomolecular | $\beta_{0}=K$  $\beta_{1}={K-V}_{0}$  $\beta_{2}=\alpha$ | $\log V\left( t \right)={log(\beta}_{0}-\beta_{1}e^{-\beta_{2}t})$ | $\beta_{0}=1000$  $\beta_{1}=1000$  $\beta_{2}=0.1$ |
| Von Bertalanffy | $\beta_{0}=\left( \frac{\eta}{\kappa} \right)^{\frac{1}{1-m}}$  $\beta_{1}=1-\left( \frac{V_{0}}{A} \right)^{1-m}$  $\beta_{2}=\left( 1-m \right)\kappa$  $\beta_{3}=m$ | $\log V(t)=\log\beta_{0}+\frac{1}{1-\beta_{3}}\log\left( 1-\beta_{1}e^{-\beta_{2}t} \right)$ | $\log\beta_{0}=1$  $\beta_{1}=10$  $\beta_{2}=0.1$  $\beta_{3}=1$ |

**Supplementary Table 4. Likelihood functions of ordinary least squares and transformed response variables**

| Statistical model | Likelihood function of V |
| --- | --- |
| $V_{ij}\sim N(f\left( t_{j},\boldsymbol{\beta} \right), \sigma^{2})$ | $f\left( V_{ij} \right\vert f\left( t_{j},\boldsymbol{\beta} \right), \sigma)=\frac{1}{\sqrt{2\pi}\sigma}\exp[-\frac{1}{2}\frac{\left[ V_{ij}-f\left( t_{j},\boldsymbol{\beta} \right) \right]^{2}}{\sigma^{2}}]$ |
| $log(V_{ij})\sim N(\log(f\left( t_{j},\boldsymbol{\beta} \right)), \sigma^{2})$ | $f\left( V_{ij} \right\vert f\left( t_{j},\boldsymbol{\beta} \right), \sigma)=\frac{1}{V\sqrt{2\pi}\sigma}\exp[-\frac{1}{2}\frac{\left[ {log(V}_{ij})-log(f\left( t_{j},\boldsymbol{\beta} \right)) \right]^{2}}{\sigma^{2}}]$ |
| $V_{ij}^{\frac{1}{4}}\sim N\left( f^{\frac{1}{4}}\left( t_{j},\boldsymbol{\beta} \right), \sigma^{2} \right)$ | $f\left( V_{ij} \right\vert f\left( t_{j},\boldsymbol{\beta} \right), \sigma)=\frac{1}{4V^{\frac{3}{4}}\sqrt{2\pi}\sigma}\exp[-\frac{1}{2}\frac{\left[ V_{ij}^{\frac{1}{4}}-f^{\frac{1}{4}}\left( t_{j},\boldsymbol{\beta} \right) \right]^{2}}{\sigma^{2}}]$ |
| $V_{ij}^{-\frac{1}{4}}\sim N(f^{-\frac{1}{4}}\left( t_{j},\boldsymbol{\beta} \right), \sigma^{2})$ | $f\left( V_{ij} \right\vert f\left( t_{j},\boldsymbol{\beta} \right), \sigma)=\frac{1}{4V^{\frac{5}{4}}\sqrt{2\pi}\sigma}\exp[-\frac{1}{2}\frac{\left[ V_{ij}^{-\frac{1}{4}}-f^{-\frac{1}{4}}\left( t_{j},\boldsymbol{\beta} \right) \right]^{2}}{\sigma^{2}}]$ |

**Supplementary Table 5. Proportion of AIC values that become smaller after log transformation of tumor volume for each parametric model.**

| Model | Proportion |
| --- | --- |
| Exponential | 0.908 |
| Exponential square | 0.92 |
| Gompertz | 0.926 |
| Logistic | 0.924 |
| Monomolecular | 0.927 |
| Von Bertalanffy | 0.923 |

**Supplementary Table 6. Summary statistics of ΔAIC (AIC_log-transformed-TV_ – AIC_TV_)**

| Statistic | Expo | Expo.quad | Gompertz | Logistic | Mono | Bert |
| --- | --- | --- | --- | --- | --- | --- |
| 1st Qu.^a^ | -61.7 | -67.7 | -68.2 | -67.4 | -62.6 | -68.3 |
| 3rd Qu. | -10.2 | -12.5 | -13.0 | -12.8 | -12.5 | -13.0 |
| Max. | 192 | 198 | 198 | 197 | 199 | 198 |
| Mean | -48.2 | -52.6 | -52.2 | -51.7 | -49.8 | -52.2 |
| Median | -30.6 | -35.4 | -35.9 | -35.3 | -33.6 | -36.1 |
| Min. | -2082 | -2208 | -2079 | -2079 | -2083 | -2078 |

^a^Qu. stands for quantile.

**Supplementary Table 7. Lack-of-fit F-test result for each parametric model**

|  | **Exponential** | **Exponential quadratic** | **Gompertz** | **Logistic** | **Monomolecular** | **von Bertalanffy** |
| --- | --- | --- | --- | --- | --- | --- |
| Percent of lack of fit (p-val < 0.05) | 37.3% | 12.2% | 12.8% | 15.3% | 44.4% | 12.4% |

**Supplementary Table 8. Proportion of K-L best model confidence sets that contain each parametric model for each case study.**

| Model | Paclitaxel | Anti-PD-1 | Cetuximab | Irinotecan | Sorafenib |
| --- | --- | --- | --- | --- | --- |
| Exponential | 80 | 129 | 44 | 87 | 45 |
| Exponential quadratic | 102 | 238 | 54 | 105 | 54 |
| Gompertz | 100 | 245 | 52 | 101 | 55 |
| Logistic | 100 | 235 | 51 | 102 | 54 |
| Monomolecular | 64 | 89 | 37 | 90 | 42 |
| von Bertalanffy | 100 | 250 | 51 | 101 | 56 |
| Total number of experimental groups | 102 | 252 | 54 | 106 | 56 |
