# Supplementary material for: Mathematical Modeling of Tumor Growth in Preclinical Mouse Models with Applications in Biomarker Discovery and Drug Mechanism Studies
Source: Cancer Res Commun. 2024 Aug 29;4(8):2267–81. doi: 10.1158/2767-9764.CRC-24-0059 (PMC11360417; doi:10.1158/2767-9764.CRC-24-0059)
Supplement: Figure S1 [file crc-24-0059_figure_s1_supps1.pdf]

Fig. S1

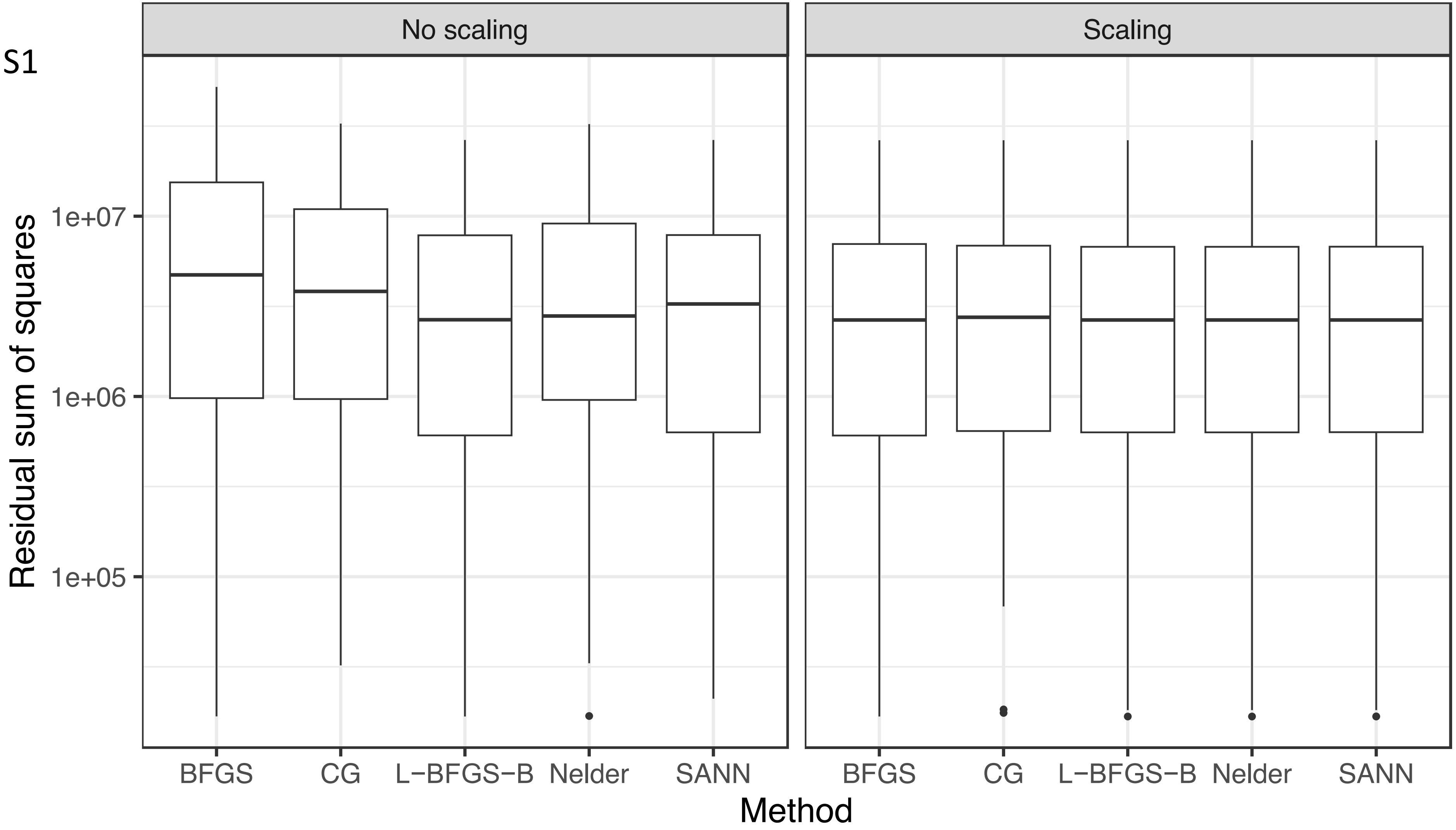

Supplementary Figure 1. Residual sum of squares of 100 randomly selected experimental groups fitted with and without parameter scaling using 5 optimization methods: Nelder-Mead, BFGS, CG, L-BFGS-B, and SANN. 5 optimization methods achieved almost the same residual sum of squares after parameter scaling.
