# Supplementary figures and images for: Mathematical Modeling of Tumor Growth in Preclinical Mouse Models with Applications in Biomarker Discovery and Drug Mechanism Studies
Source: Cancer Res Commun. 2024 Aug 29;4(8):2267–81. doi: 10.1158/2767-9764.CRC-24-0059 (PMC11360417; doi:10.1158/2767-9764.CRC-24-0059)

Fig. S2

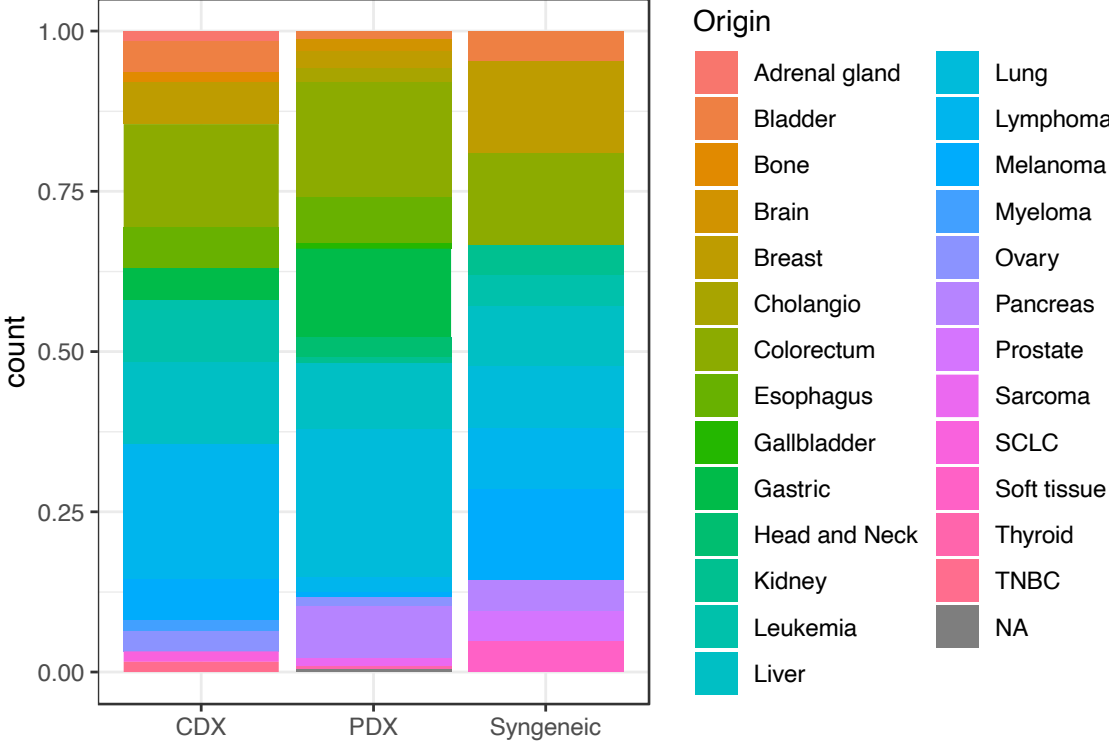

Supplementary Figure 2. Proportion of cancer origins for each mouse model type.

Supplement: Figure S2 [file crc-24-0059_figure_s2_supps2.pdf]

Fig. S4

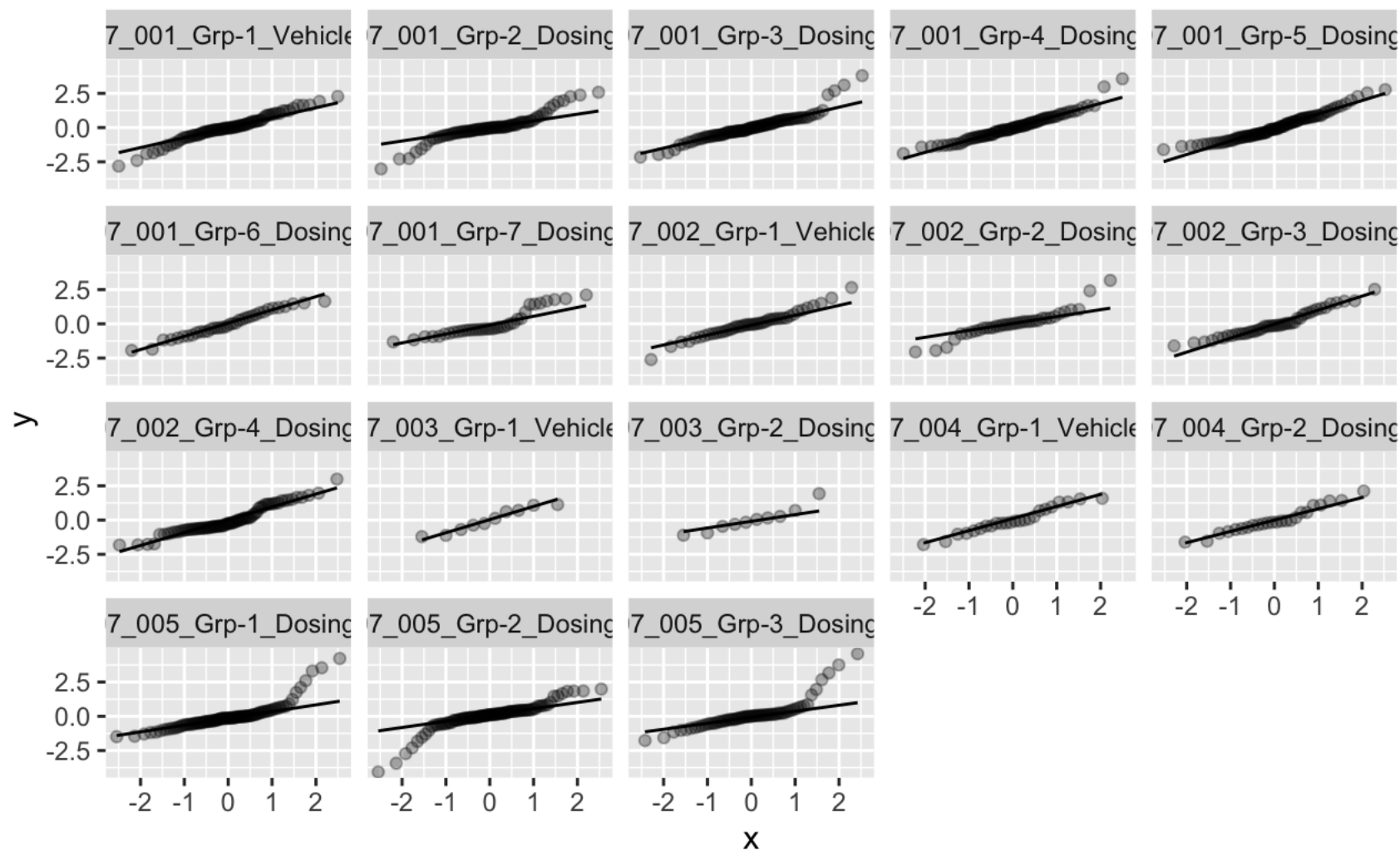

Supplementary Figure 4. Representative QQ plots of residuals fitted by exponential model.

Supplement: Figure S4 [file crc-24-0059_figure_s4_supps4.pdf]
