# Supplementary material for: Mathematical Modeling of Tumor Growth in Preclinical Mouse Models with Applications in Biomarker Discovery and Drug Mechanism Studies
Source: Cancer Res Commun. 2024 Aug 29;4(8):2267–81. doi: 10.1158/2767-9764.CRC-24-0059 (PMC11360417; doi:10.1158/2767-9764.CRC-24-0059)
Supplement: Figure S5 [file crc-24-0059_figure_s5_supps5.pdf]

Fig. S5

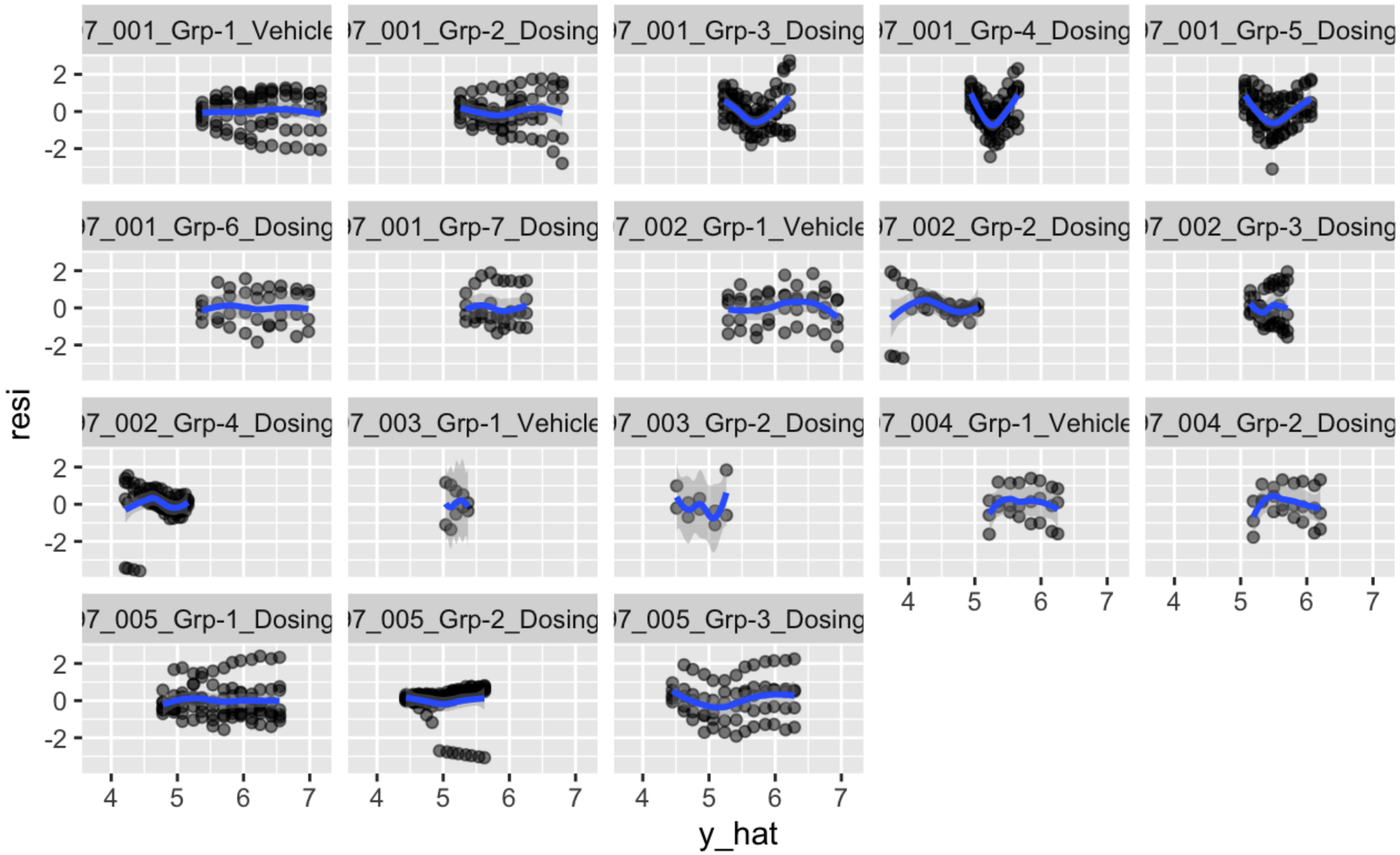

Supplementary Figure 5. Representative residual plots of linear model fitting after log transformation of tumor volume, with standardized residuals on y axis and fitted log tumor volumes on x axis. Blue line is loess regression curve.
