# Supplementary material for: Mathematical Modeling of Tumor Growth in Preclinical Mouse Models with Applications in Biomarker Discovery and Drug Mechanism Studies
Source: Cancer Res Commun. 2024 Aug 29;4(8):2267–81. doi: 10.1158/2767-9764.CRC-24-0059 (PMC11360417; doi:10.1158/2767-9764.CRC-24-0059)
Supplement: Figure S8 [file crc-24-0059_figure_s8_supps8.pdf]

Fig. S8

A

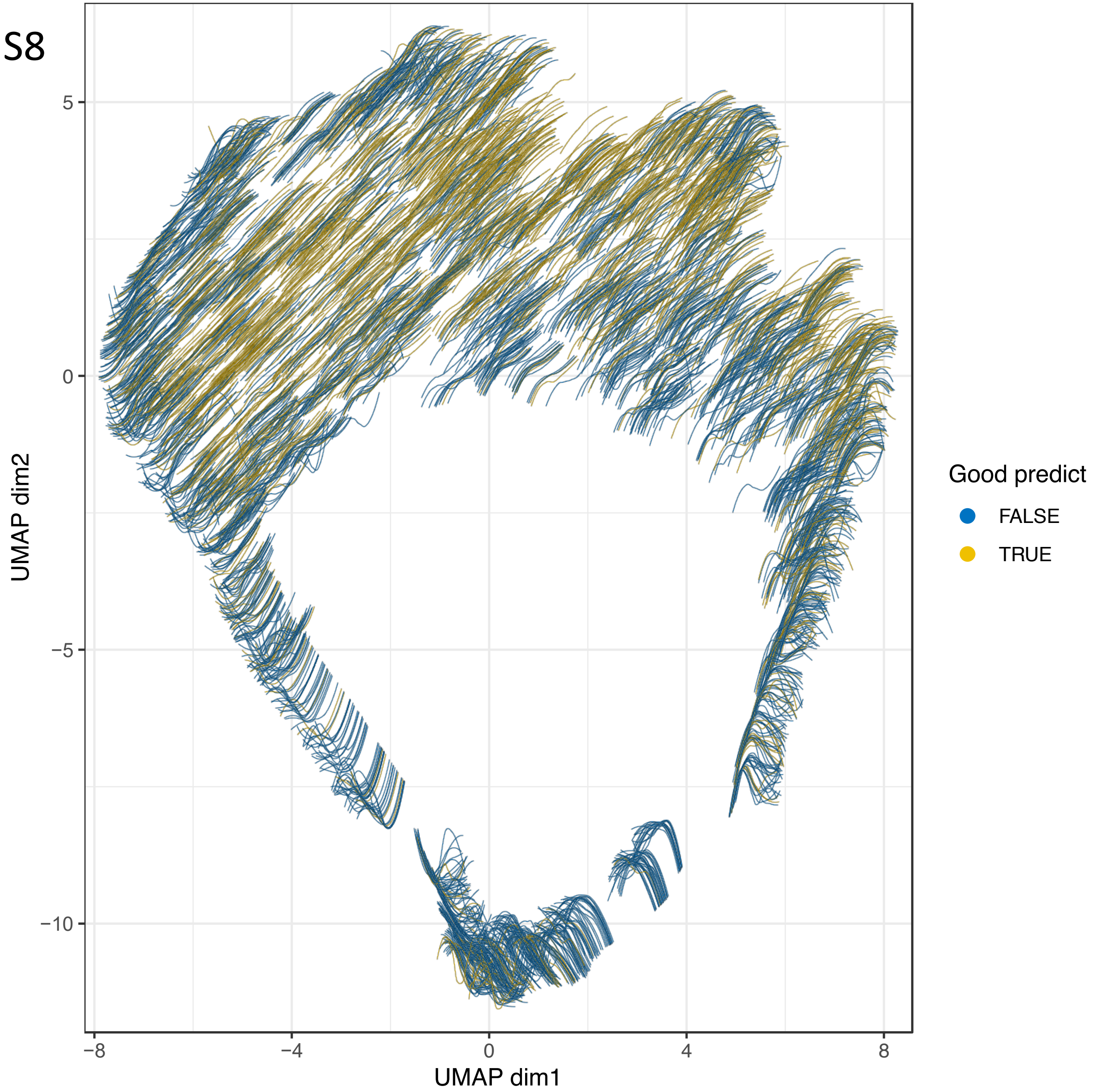

B

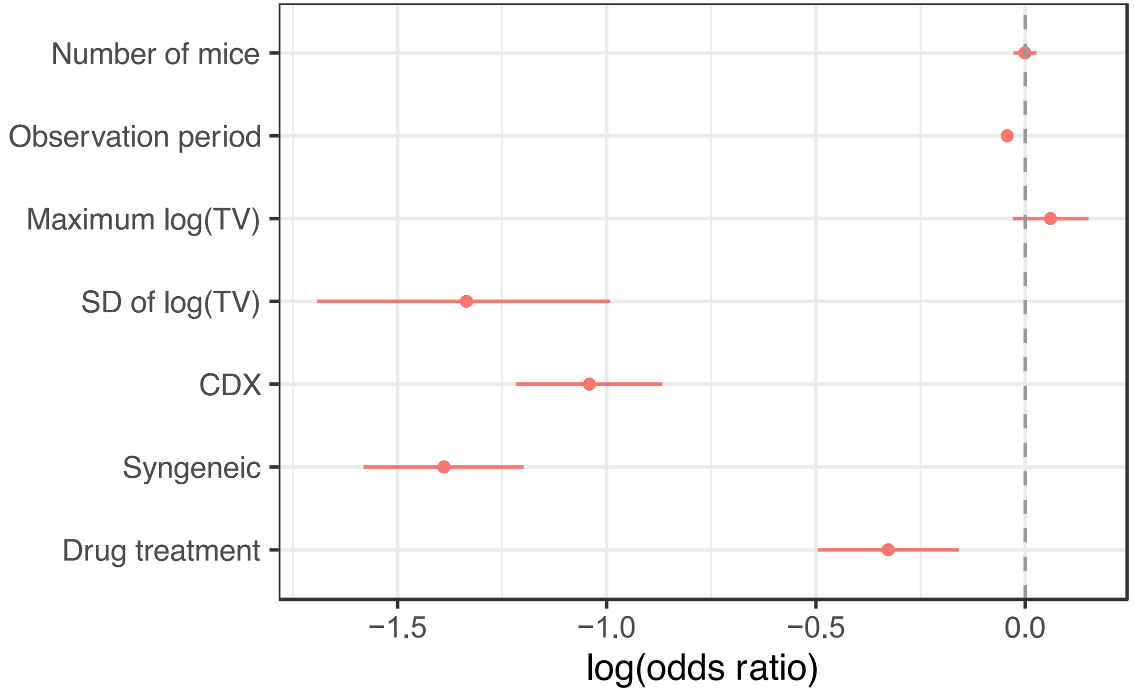

Supplementary Figure 8. Factors related to predictability. A) UMAP projection of all curves. “Good prediction” (defined as last time point prediction deviate from actual mean tumor volume by less than 25% for exponential quardratic, Gompertz, logistic, and von Bertalanffy) are labeled yellow. B) Forest plot of logistic regression of factors on “good prediction”. Point stands for average log(odds ratio) and range stands for 95% confidence interval.
