# Supplementary material for: Mathematical Modeling of Tumor Growth in Preclinical Mouse Models with Applications in Biomarker Discovery and Drug Mechanism Studies
Source: Cancer Res Commun. 2024 Aug 29;4(8):2267–81. doi: 10.1158/2767-9764.CRC-24-0059 (PMC11360417; doi:10.1158/2767-9764.CRC-24-0059)
Supplement: Figure S15 [file crc-24-0059_figure_s15_supps15.pdf]

Fig. S15A

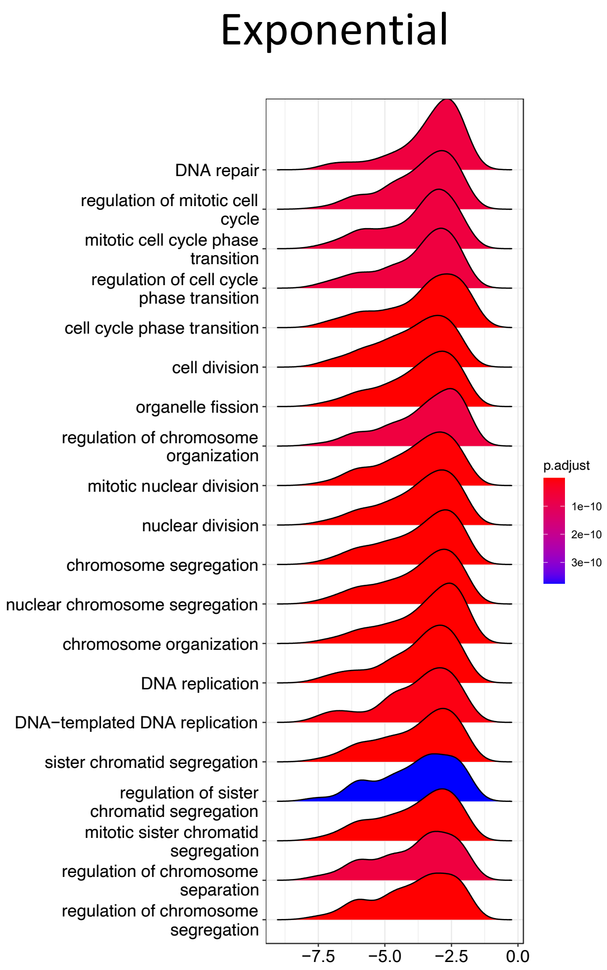

Exponential
DNA repair
regulation of mitotic cell cycle
mitotic cell cycle phase transition
regulation of cell cycle phase transition
cell cycle phase transition
cell division
organelle fission
regulation of chromosome organization
mitotic nuclear division
nuclear division
chromosome segregation
nuclear chromosome segregation
chromosome organization
DNA replication
DNA−templated DNA replication
sister chromatid segregation
regulation of sister chromatid segregation
mitotic sister chromatid segregation
regulation of chromosome separation
regulation of chromosome segregation
−7.5
−5.0
−2.5
0.0
p.adjust
1e−10
2e−10
3e−10

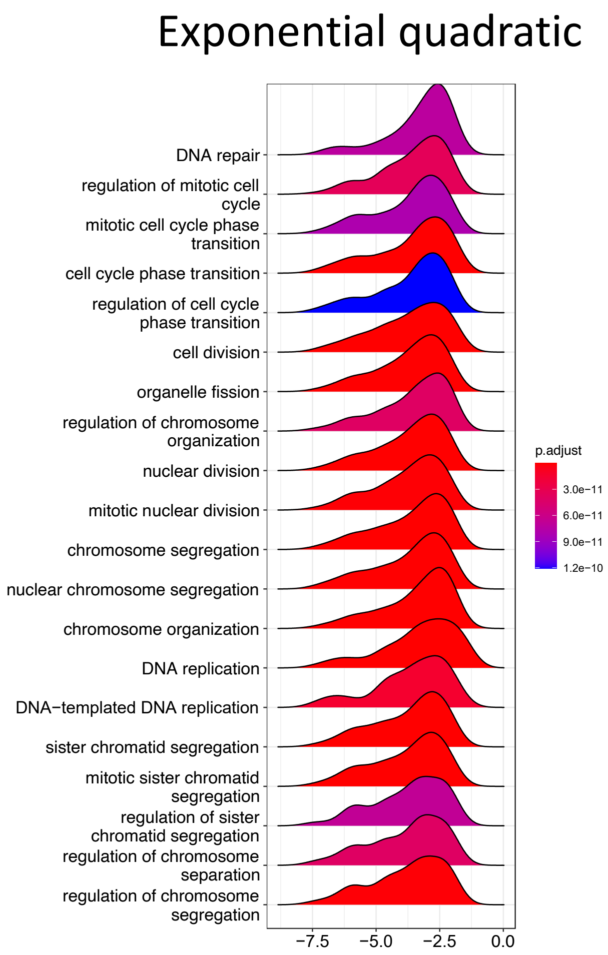

Exponential quadratic
DNA repair
regulation of mitotic cell cycle
mitotic cell cycle phase transition
cell cycle phase transition
regulation of cell cycle phase transition
cell division
organelle fission
regulation of chromosome organization
nuclear division
mitotic nuclear division
chromosome segregation
nuclear chromosome segregation
chromosome organization
DNA replication
DNA−templated DNA replication
sister chromatid segregation
mitotic sister chromatid segregation
regulation of sister chromatid segregation
regulation of chromosome separation
regulation of chromosome segregation
−7.5
−5.0
−2.5
0.0
p.adjust
3.0e−11
6.0e−11
9.0e−11
1.2e−10

Fig. S15B

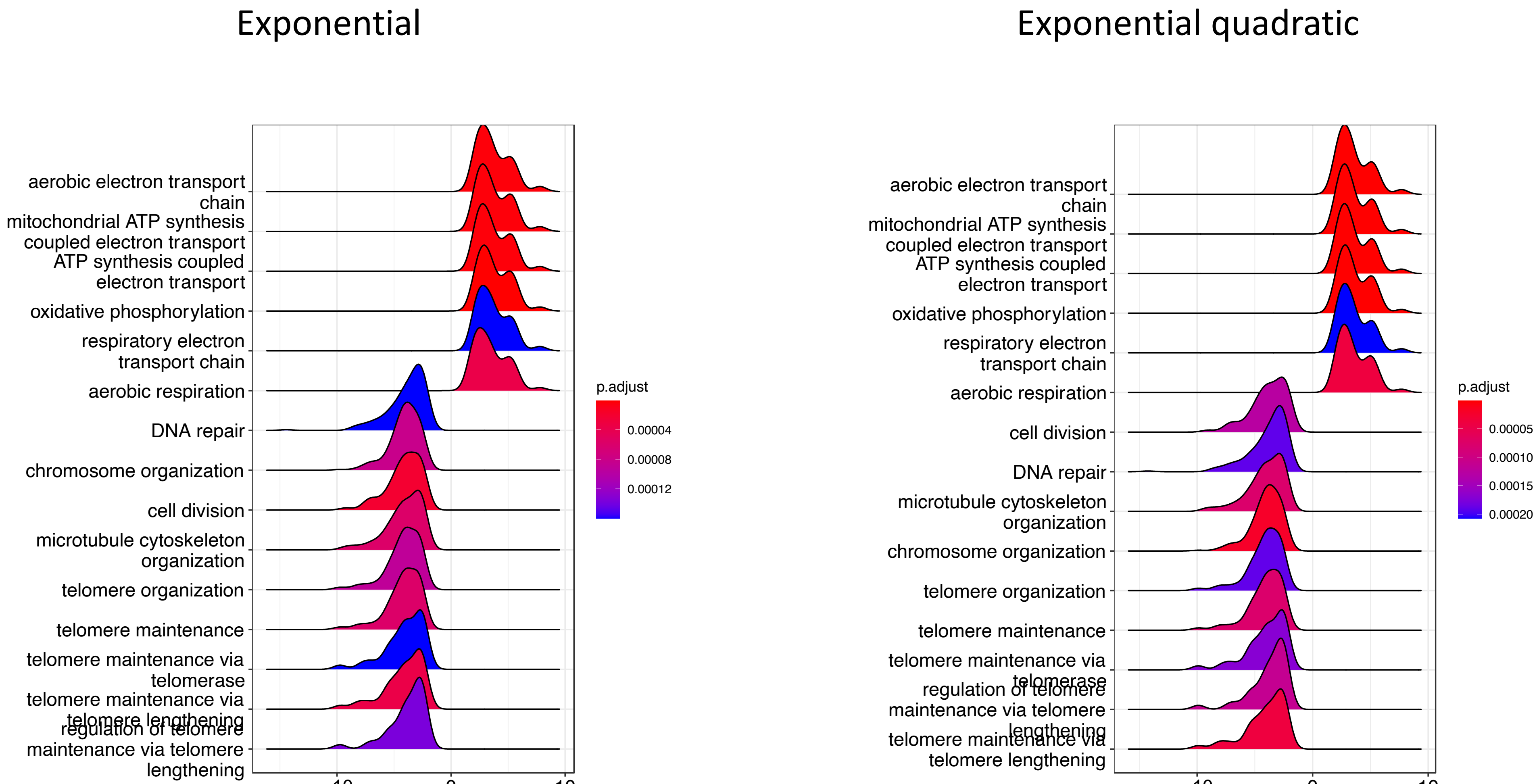
Exponential
Exponential quadratic
aerobic electron transport chain
mitochondrial ATP synthesis coupled electron transport
ATP synthesis coupled electron transport
oxidative phosphorylation
respiratory electron transport chain
aerobic respiration
DNA repair
chromosome organization
cell division
microtubule cytoskeleton organization
telomere organization
telomere maintenance
telomere maintenance via telomerase
telomere maintenance via telomere lengthening
regulation of telomere maintenance via telomere lengthening
p.adjust
0.00004
0.00008
0.00012
0.00005
0.00010
0.00015
0.00020
−10
0
10

Fig. S15C

Exponential

Exponential quadratic

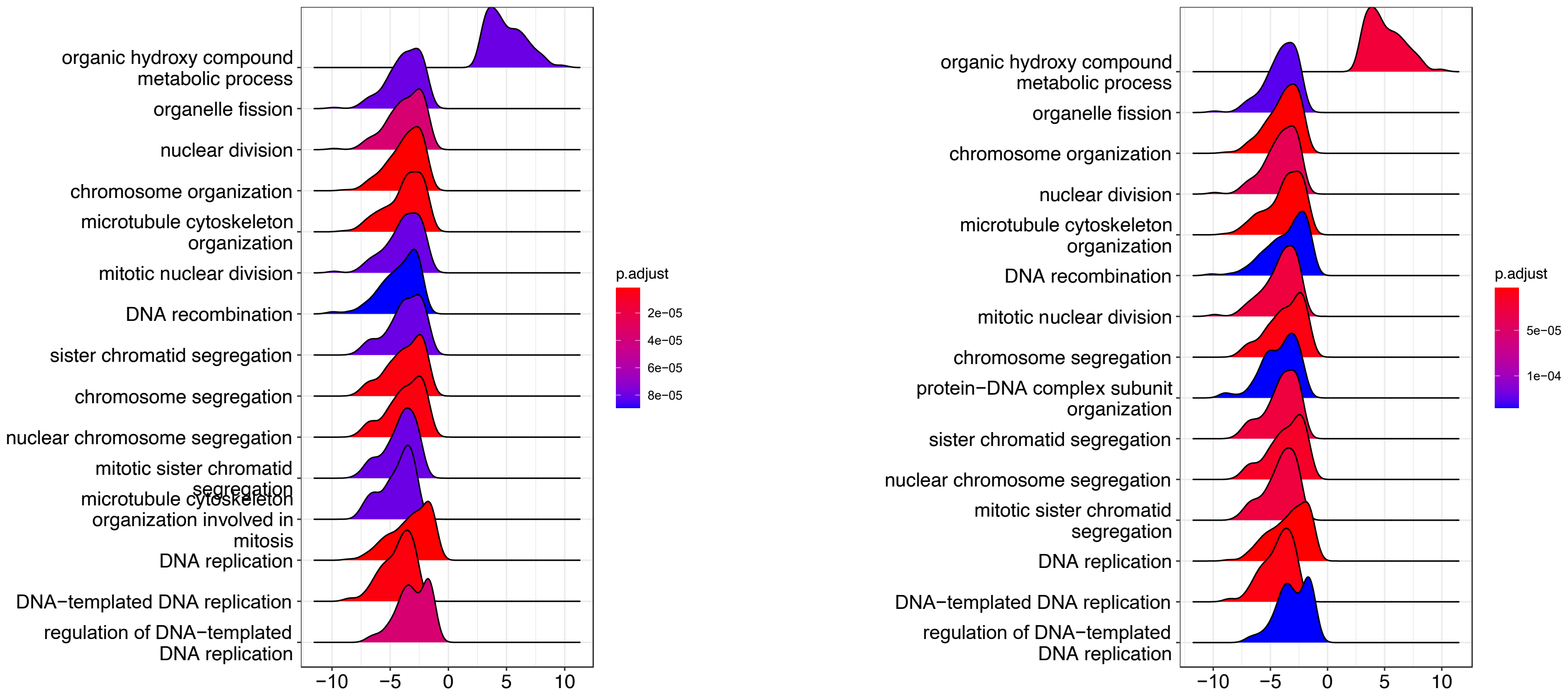

Fig. S15D

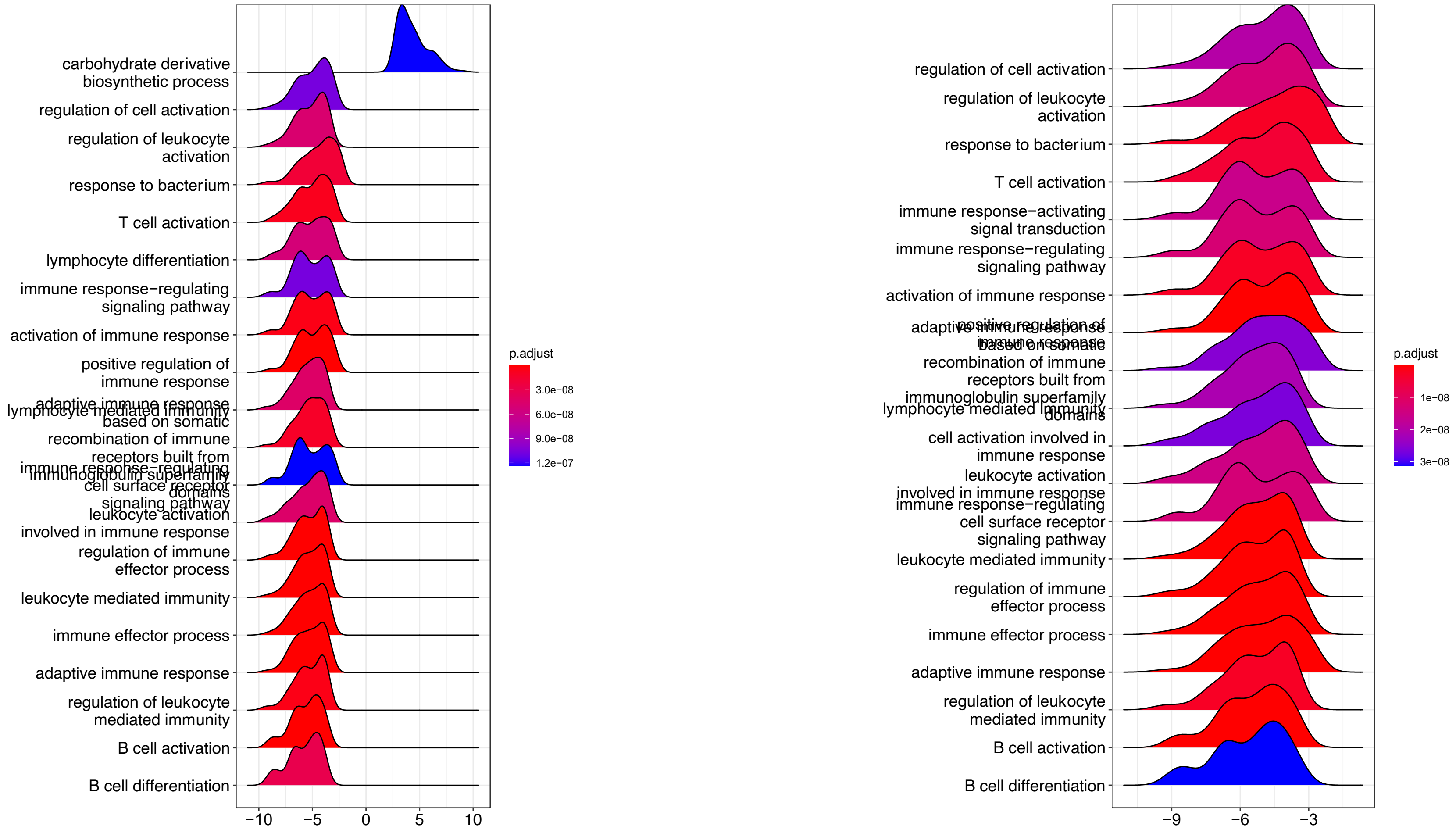
carbohydrate derivative biosynthetic process
regulation of cell activation
regulation of leukocyte activation
response to bacterium
T cell activation
lymphocyte differentiation
immune response−regulating signaling pathway
activation of immune response
positive regulation of immune response
adaptive immune response based on somatic recombination of immune receptors built from immunoglobulin superfamily domains
lymphocyte mediated immunity
immune response−regulating cell surface receptor signaling pathway
leukocyte activation involved in immune response
regulation of immune effector process
leukocyte mediated immunity
immune effector process
adaptive immune response
regulation of leukocyte mediated immunity
B cell activation
B cell differentiation
−10
−5
0
5
10
p.adjust
3.0e−08
6.0e−08
9.0e−08
1.2e−07
regulation of cell activation
regulation of leukocyte activation
response to bacterium
T cell activation
immune response−activating signal transduction
immune response−regulating signaling pathway
activation of immune response
positive regulation of immune response
adaptive immune response based on somatic recombination of immune receptors built from immunoglobulin superfamily domains
lymphocyte mediated immunity
cell activation involved in immune response
leukocyte activation involved in immune response
immune response−regulating cell surface receptor signaling pathway
leukocyte mediated immunity
regulation of immune effector process
immune effector process
adaptive immune response
regulation of leukocyte mediated immunity
B cell activation
B cell differentiation
−9
−6
−3
p.adjust
1e−08
2e−08
3e−08

Fig. S15E

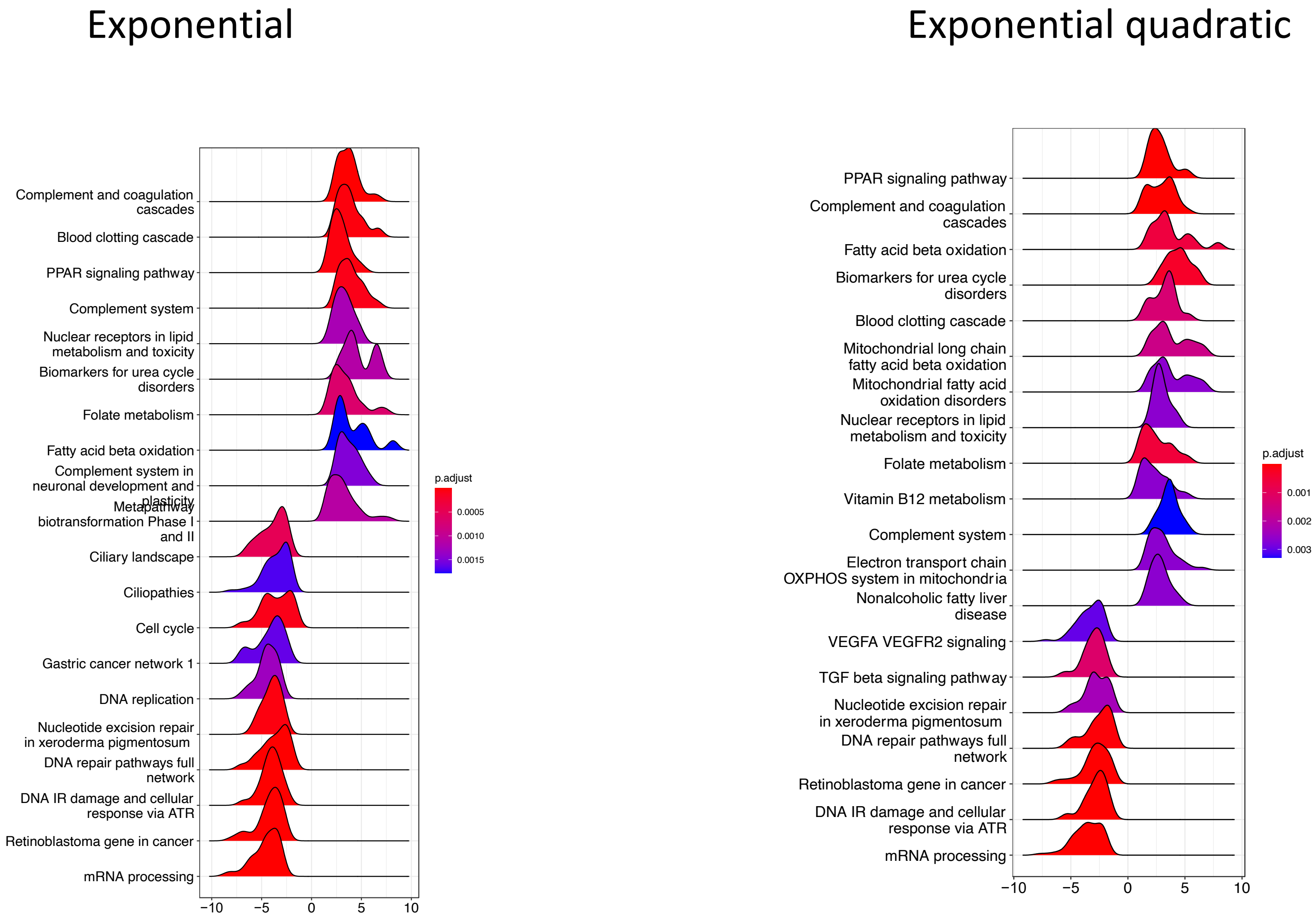

Supplementary Figure 15. Compare GSEA between exponential and exponential quadratic model (Genes ordered by descending t-value of Day:Gene:Treatment) A) Irinotecan data set (GO Biologcial Process (BP)). B) Cetuximab data set (GO_BP). Both identified “positive regulation of ERBB signaling pathway”, but not shown in the plots. C) Paclitaxel data set (GO BP). D) PD1 data set (GO BP). E) Sorafenib data set (Wikipathways).
