# Supplementary material for: Mathematical Modeling of Tumor Growth in Preclinical Mouse Models with Applications in Biomarker Discovery and Drug Mechanism Studies
Source: Cancer Res Commun. 2024 Aug 29;4(8):2267–81. doi: 10.1158/2767-9764.CRC-24-0059 (PMC11360417; doi:10.1158/2767-9764.CRC-24-0059)
Supplement: Figure S16 [file crc-24-0059_figure_s16_supps16.pdf]

Fig. S16A

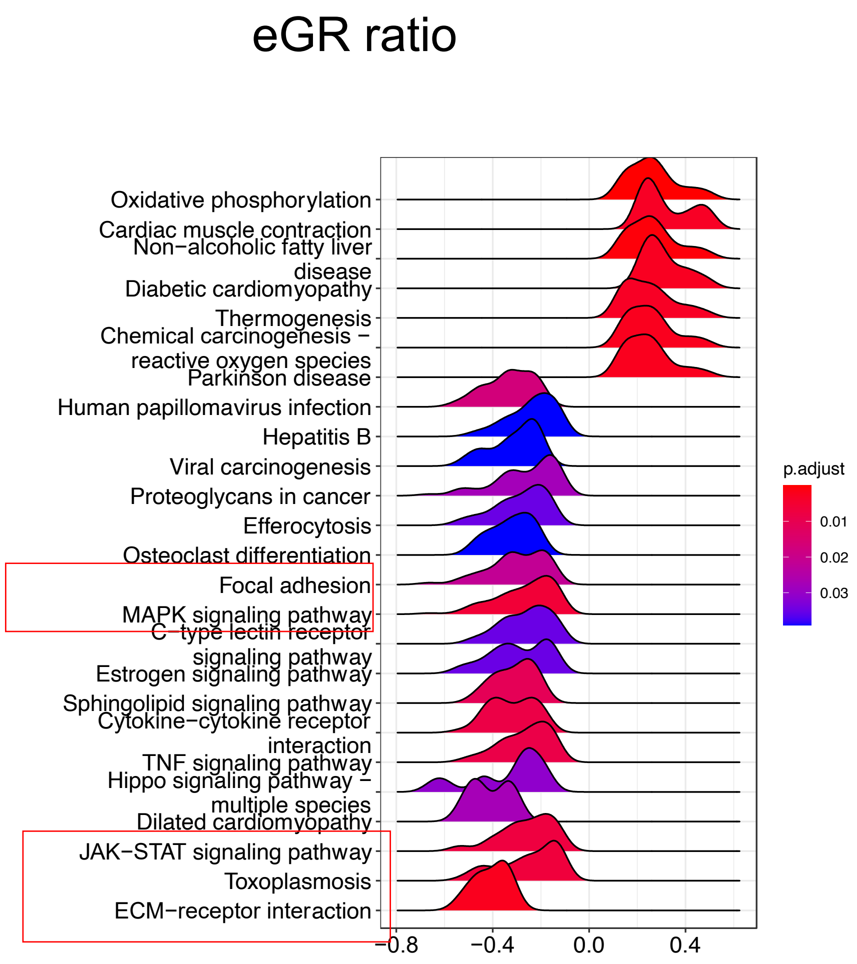
eGR ratio
Oxidative phosphorylation
Cardiac muscle contraction
Non−alcoholic fatty liver disease
Diabetic cardiomyopathy
Thermogenesis
Chemical carcinogenesis − reactive oxygen species
Parkinson disease
Human papillomavirus infection
Hepatitis B
Viral carcinogenesis
Proteoglycans in cancer
Efferocytosis
Osteoclast differentiation
Focal adhesion
MAPK signaling pathway
C−type lectin receptor signaling pathway
Estrogen signaling pathway
Sphingolipid signaling pathway
Cytokine−cytokine receptor interaction
TNF signaling pathway
Hippo signaling pathway − multiple species
Dilated cardiomyopathy
JAK−STAT signaling pathway
Toxoplasmosis
ECM−receptor interaction
−0.8
−0.4
0.0
0.4
p.adjust
0.01
0.02
0.03

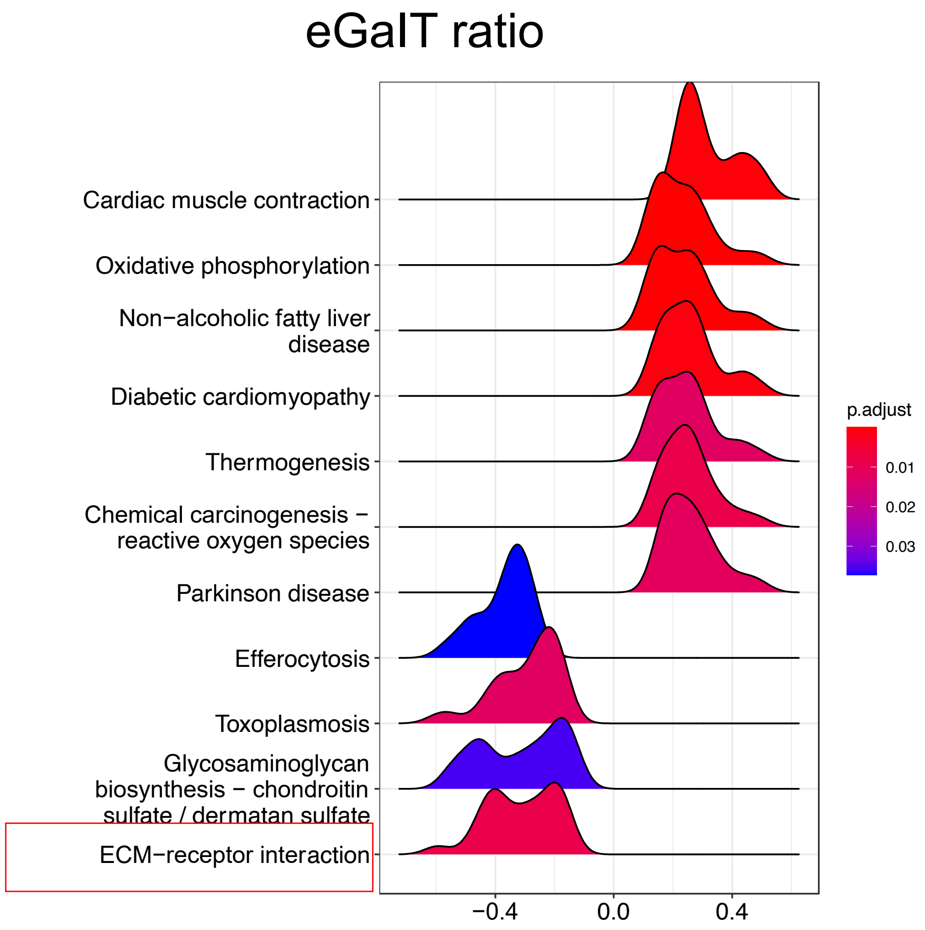
eGalT ratio
Cardiac muscle contraction
Oxidative phosphorylation
Non−alcoholic fatty liver disease
Diabetic cardiomyopathy
Thermogenesis
Chemical carcinogenesis − reactive oxygen species
Parkinson disease
Efferocytosis
Toxoplasmosis
Glycosaminoglycan biosynthesis − chondroitin sulfate / dermatan sulfate
ECM−receptor interaction
−0.4
0.0
0.4
p.adjust
0.01
0.02
0.03

Fig. S16B

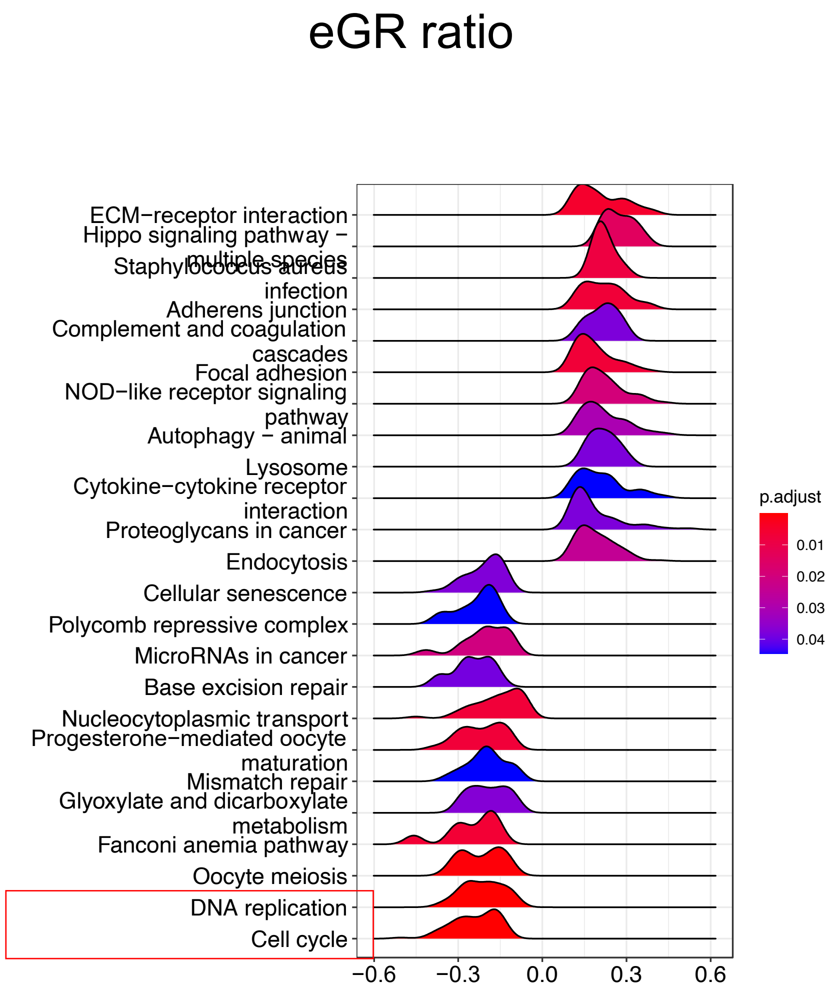

eGR ratio
ECM−receptor interaction
Hippo signaling pathway − multiple species
Staphylococcus aureus infection
Adherens junction
Complement and coagulation cascades
Focal adhesion
NOD−like receptor signaling pathway
Autophagy − animal
Lysosome
Cytokine−cytokine receptor interaction
Proteoglycans in cancer
Endocytosis
Cellular senescence
Polycomb repressive complex
MicroRNAs in cancer
Base excision repair
Nucleocytoplasmic transport
Progesterone−mediated oocyte maturation
Mismatch repair
Glyoxylate and dicarboxylate metabolism
Fanconi anemia pathway
Oocyte meiosis
DNA replication
Cell cycle
−0.6 −0.3 0.0 0.3 0.6
p.adjust
0.01
0.02
0.03
0.04

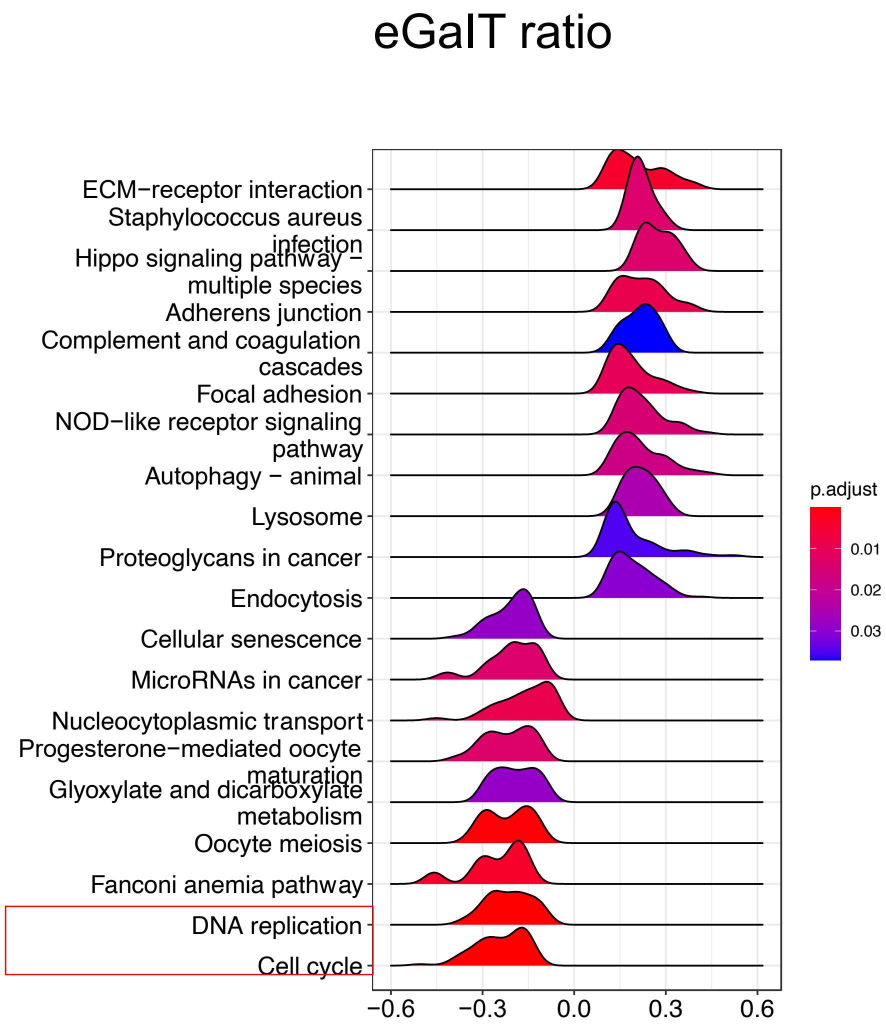

eGalT ratio
ECM−receptor interaction
Staphylococcus aureus infection
Hippo signaling pathway − multiple species
Adherens junction
Complement and coagulation cascades
Focal adhesion
NOD−like receptor signaling pathway
Autophagy − animal
Lysosome
Proteoglycans in cancer
Endocytosis
Cellular senescence
MicroRNAs in cancer
Nucleocytoplasmic transport
Progesterone−mediated oocyte maturation
Glyoxylate and dicarboxylate metabolism
Oocyte meiosis
Fanconi anemia pathway
DNA replication
Cell cycle
−0.6 −0.3 0.0 0.3 0.6
p.adjust
0.01
0.02
0.03

Fig. S16C

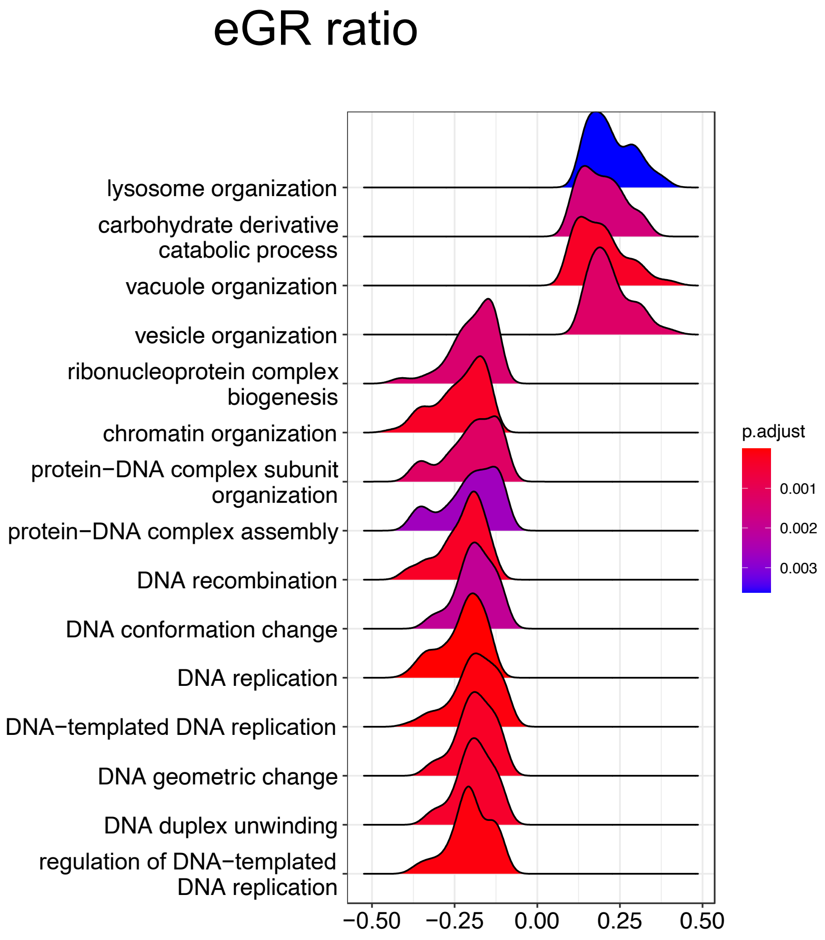
eGR ratio
lysosome organization
carbohydrate derivative catabolic process
vacuole organization
vesicle organization
ribonucleoprotein complex biogenesis
chromatin organization
protein−DNA complex subunit organization
protein−DNA complex assembly
DNA recombination
DNA conformation change
DNA replication
DNA−templated DNA replication
DNA geometric change
DNA duplex unwinding
regulation of DNA−templated DNA replication
−0.50
−0.25
0.00
0.25
0.50
p.adjust
0.001
0.002
0.003

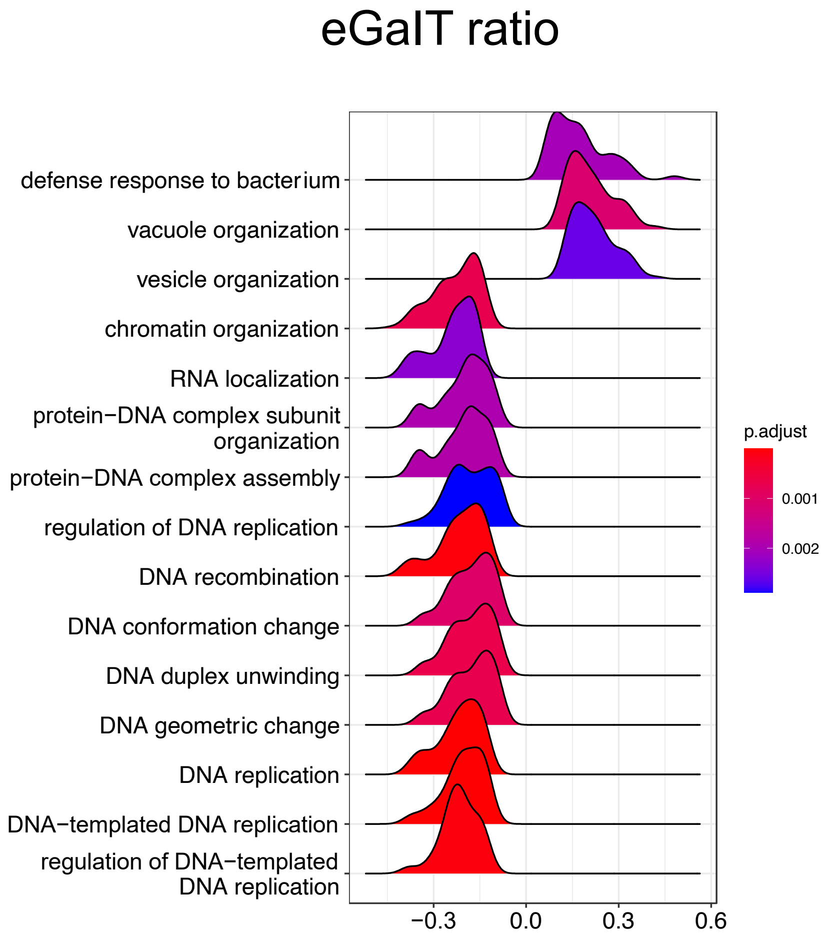
eGaIT ratio
defense response to bacterium
vacuole organization
vesicle organization
chromatin organization
RNA localization
protein−DNA complex subunit organization
protein−DNA complex assembly
regulation of DNA replication
DNA recombination
DNA conformation change
DNA duplex unwinding
DNA geometric change
DNA replication
DNA−templated DNA replication
regulation of DNA−templated DNA replication
−0.3
0.0
0.3
0.6
p.adjust
0.001
0.002

Fig. S16D

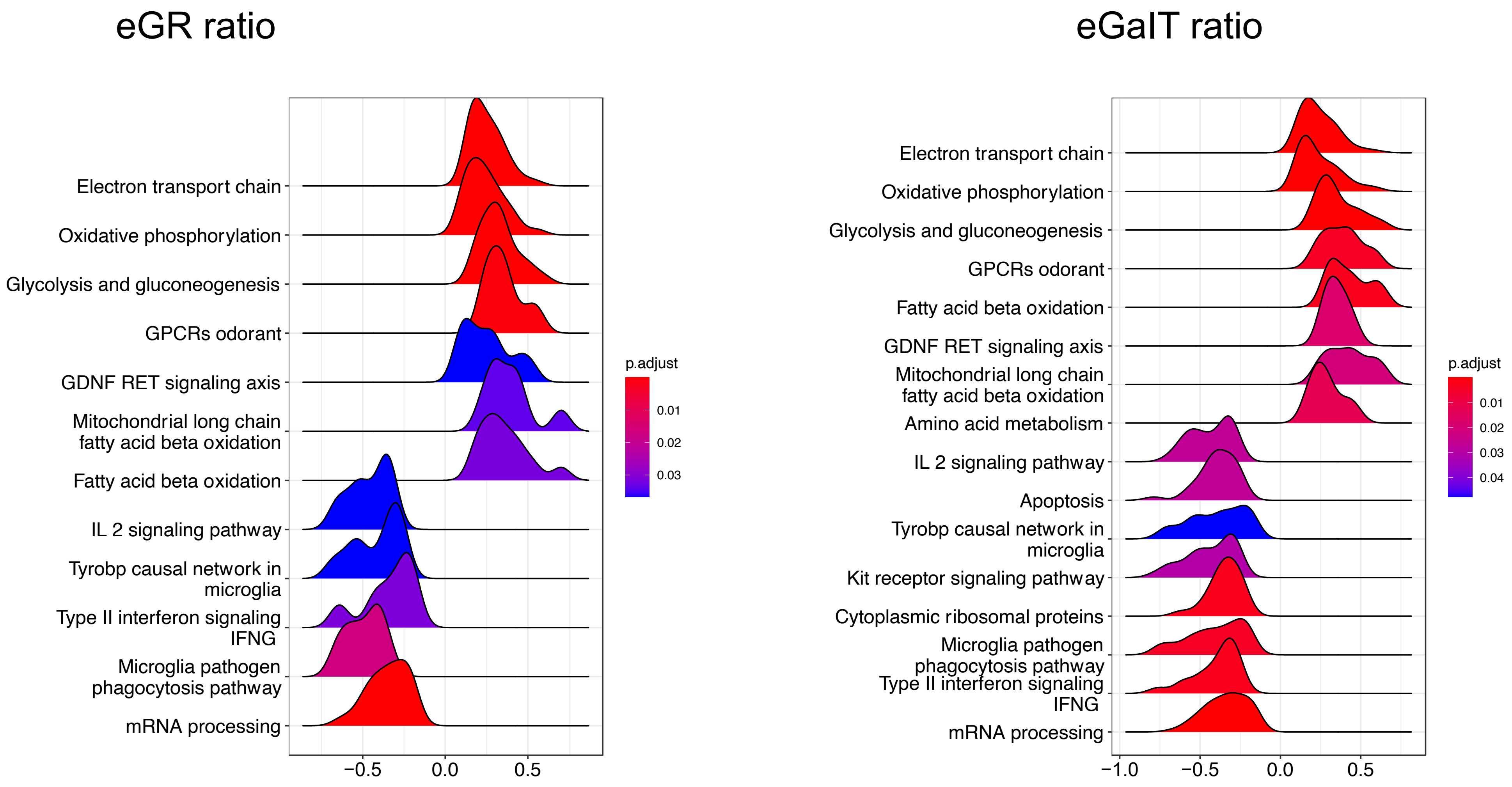
eGR ratio
Electron transport chain
Oxidative phosphorylation
Glycolysis and gluconeogenesis
GPCRs odorant
GDNF RET signaling axis
Mitochondrial long chain fatty acid beta oxidation
Fatty acid beta oxidation
IL 2 signaling pathway
Tyrobp causal network in microglia
Type II interferon signaling IFNG
Microglia pathogen phagocytosis pathway
mRNA processing
−0.5
0.0
0.5
p.adjust
0.01
0.02
0.03
eGaIT ratio
Electron transport chain
Oxidative phosphorylation
Glycolysis and gluconeogenesis
GPCRs odorant
Fatty acid beta oxidation
GDNF RET signaling axis
Mitochondrial long chain fatty acid beta oxidation
Amino acid metabolism
IL 2 signaling pathway
Apoptosis
Tyrobp causal network in microglia
Kit receptor signaling pathway
Cytoplasmic ribosomal proteins
Microglia pathogen phagocytosis pathway
Type II interferon signaling IFNG
mRNA processing
−1.0
−0.5
0.0
0.5
p.adjust
0.01
0.02
0.03
0.04

Fig. S16E

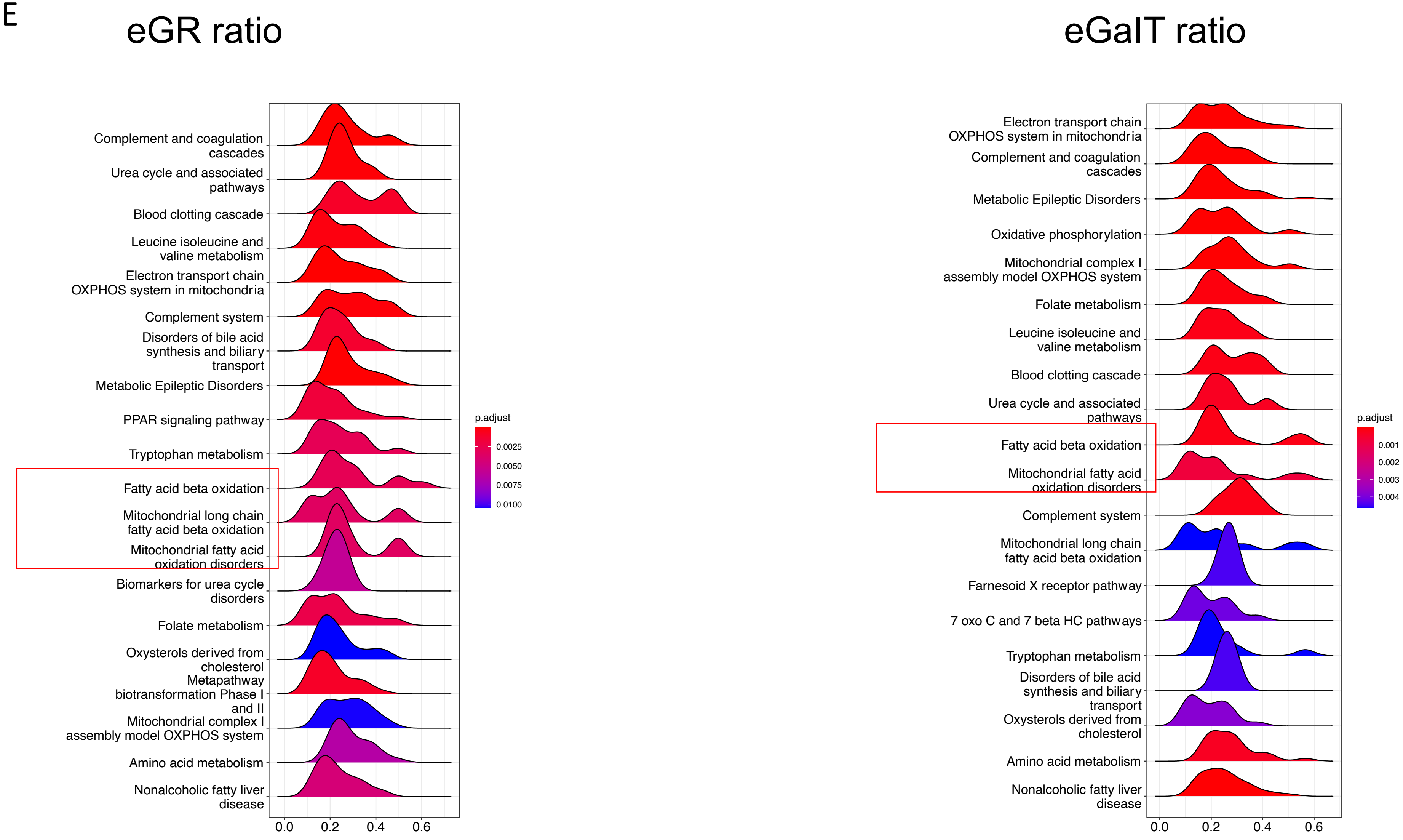

Supplementary Figure 16. Compare GSEA analysis between eGFR ratio and eGalT ratio. Genes were ordered by Spearman correlation between gene expression and eGR ratio / eGalT ratio. Red-rectangulated terms are target-related. A) Cetuximab KEGG pathways. B) Irinotecan KEGG pathways. C) Paclitaxel GO BP. D) PD-1 Wikipathways. E) Sorafenib Wikipathways.
