# Supplementary material for: Mathematical Modeling of Tumor Growth in Preclinical Mouse Models with Applications in Biomarker Discovery and Drug Mechanism Studies
Source: Cancer Res Commun. 2024 Aug 29;4(8):2267–81. doi: 10.1158/2767-9764.CRC-24-0059 (PMC11360417; doi:10.1158/2767-9764.CRC-24-0059)
Supplement: Figure S17 [file crc-24-0059_figure_s17_supps17.pdf]

Fig. S17A1

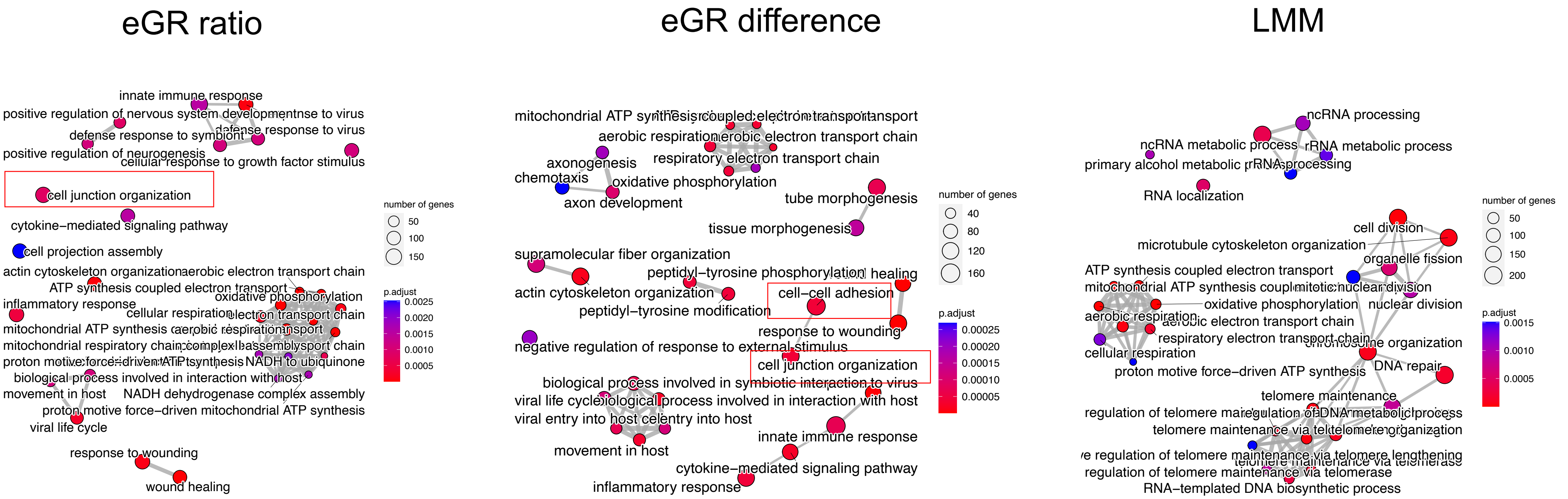
eGR ratio
innate immune response
defense response to symbiont
positive regulation of neurogenesis
cellular response to growth factor stimulus
cell junction organization
cytokine−mediated signaling pathway
cell projection assembly
actin cytoskeleton organization
ATP synthesis coupled electron transport
oxidative phosphorylation
inflammatory response
cellular respiration
NADH to ubiquinone
movement in host
NADH dehydrogenase complex assembly
proton motive force−driven mitochondrial ATP synthesis
viral life cycle
response to wounding
wound healing
number of genes
50
100
150
p.adjust
0.0025
0.0020
0.0015
0.0010
0.0005
eGR difference
aerobic electron transport chain
respiratory electron transport chain
axonogenesis
chemotaxis
oxidative phosphorylation
axon development
tube morphogenesis
tissue morphogenesis
supramolecular fiber organization
actin cytoskeleton organization
peptidyl−tyrosine modification
cell−cell adhesion
response to wounding
negative regulation of response to external stimulus
cell junction organization
innate immune response
movement in host
cytokine−mediated signaling pathway
inflammatory response
number of genes
40
80
120
160
p.adjust
0.00025
0.00020
0.00015
0.00010
0.00005
LMM
ncRNA processing
ncRNA metabolic process
rRNA metabolic process
primary alcohol metabolic process
RNA localization
cell division
microtubule cytoskeleton organization
organelle fission
ATP synthesis coupled electron transport
nuclear division
oxidative phosphorylation
aerobic respiration
cellular respiration
proton motive force−driven ATP synthesis
DNA repair
telomere maintenance
regulation of telomere maintenance via telomerase
RNA−templated DNA biosynthetic process
number of genes
50
100
150
200
p.adjust
0.0015
0.0010
0.0005

Fig. S17A2

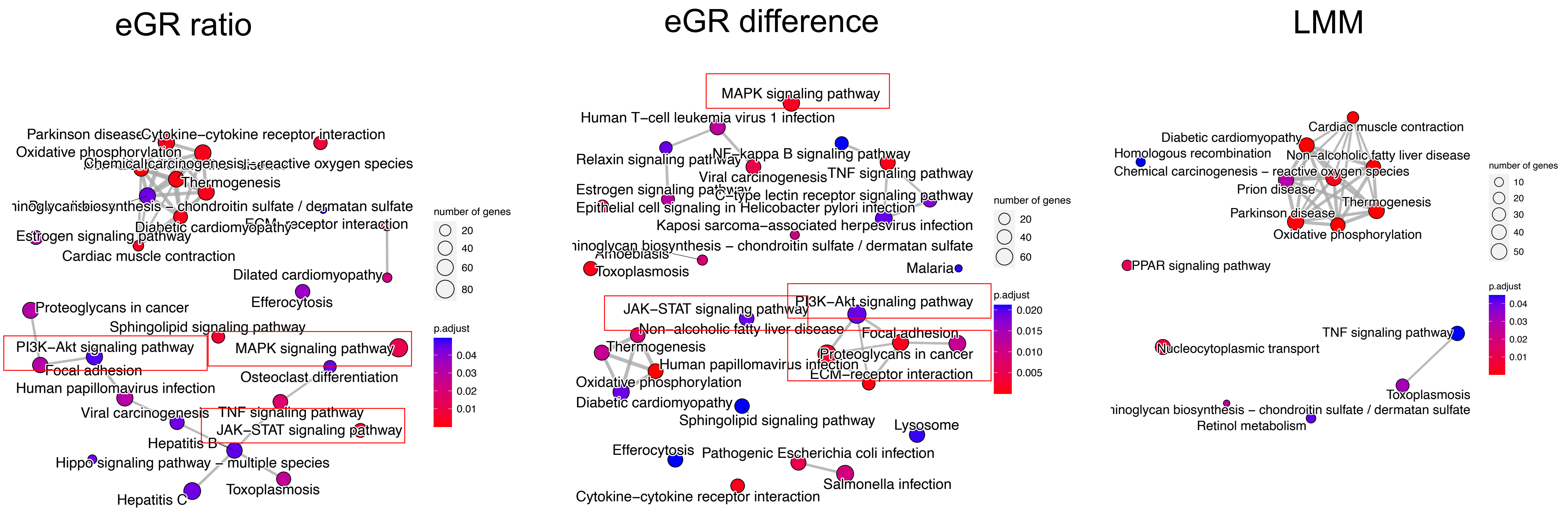
eGR ratio
Parkinson disease
Cytokine−cytokine receptor interaction
Oxidative phosphorylation
Chemical carcinogenesis − reactive oxygen species
Thermogenesis
Diabetic cardiomyopathy
ECM−receptor interaction
Estrogen signaling pathway
Cardiac muscle contraction
Dilated cardiomyopathy
Efferocytosis
Proteoglycans in cancer
Sphingolipid signaling pathway
PI3K−Akt signaling pathway
MAPK signaling pathway
Focal adhesion
Osteoclast differentiation
Human papillomavirus infection
Viral carcinogenesis
TNF signaling pathway
JAK−STAT signaling pathway
Hepatitis B
Hippo signaling pathway − multiple species
Toxoplasmosis
Hepatitis C
number of genes
20
40
60
80
p.adjust
0.04
0.03
0.02
0.01
eGR difference
MAPK signaling pathway
Human T−cell leukemia virus 1 infection
Relaxin signaling pathway
NF−kappa B signaling pathway
Viral carcinogenesis
TNF signaling pathway
Estrogen signaling pathway
C−type lectin receptor signaling pathway
Epithelial cell signaling in Helicobacter pylori infection
Kaposi sarcoma−associated herpesvirus infection
Amoebiasis
Toxoplasmosis
Malaria
JAK−STAT signaling pathway
PI3K−Akt signaling pathway
Non−alcoholic fatty liver disease
Thermogenesis
Focal adhesion
Proteoglycans in cancer
Human papillomavirus infection
ECM−receptor interaction
Oxidative phosphorylation
Diabetic cardiomyopathy
Sphingolipid signaling pathway
Lysosome
Efferocytosis
Pathogenic Escherichia coli infection
Salmonella infection
Cytokine−cytokine receptor interaction
number of genes
20
40
60
p.adjust
0.020
0.015
0.010
0.005
LMM
Cardiac muscle contraction
Diabetic cardiomyopathy
Homologous recombination
Non−alcoholic fatty liver disease
Chemical carcinogenesis − reactive oxygen species
Prion disease
Thermogenesis
Parkinson disease
Oxidative phosphorylation
PPAR signaling pathway
TNF signaling pathway
Nucleocytoplasmic transport
Toxoplasmosis
Retinol metabolism
number of genes
10
20
30
40
50
p.adjust
0.04
0.03
0.02
0.01

Fig. S17B

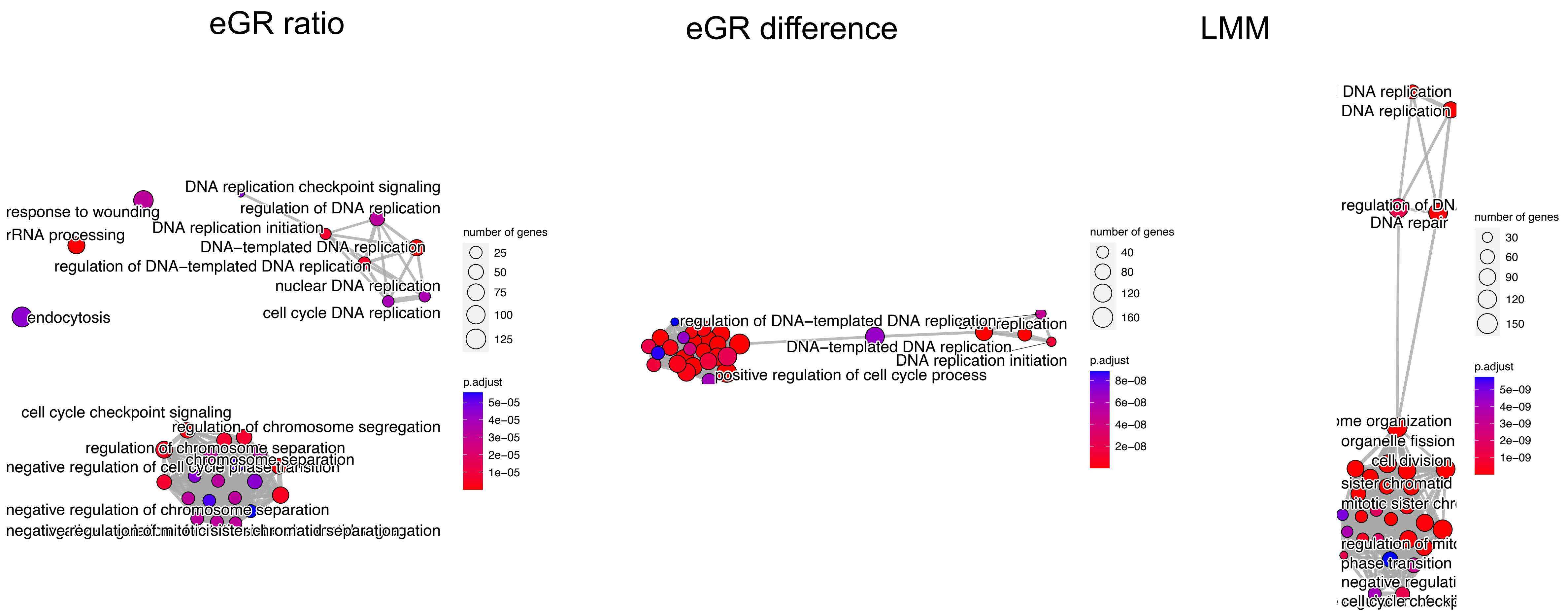
eGR ratio
eGR difference
LMM
DNA replication checkpoint signaling
regulation of DNA replication
response to wounding
DNA replication initiation
rRNA processing
DNA−templated DNA replication
regulation of DNA−templated DNA replication
nuclear DNA replication
endocytosis
cell cycle DNA replication
cell cycle checkpoint signaling
regulation of chromosome segregation
regulation of chromosome separation
chromosome separation
negative regulation of cell cycle phase transition
negative regulation of chromosome separation
number of genes
25
50
75
100
125
p.adjust
5e−05
4e−05
3e−05
2e−05
1e−05
regulation of DNA−templated DNA replication
DNA−templated DNA replication
DNA replication initiation
positive regulation of cell cycle process
number of genes
40
80
120
160
p.adjust
8e−08
6e−08
4e−08
2e−08
DNA replication
DNA replication
DNA repair
organelle fission
cell division
sister chromatid
phase transition
number of genes
30
60
90
120
150
p.adjust
5e−09
4e−09
3e−09
2e−09
1e−09

Fig. S17C

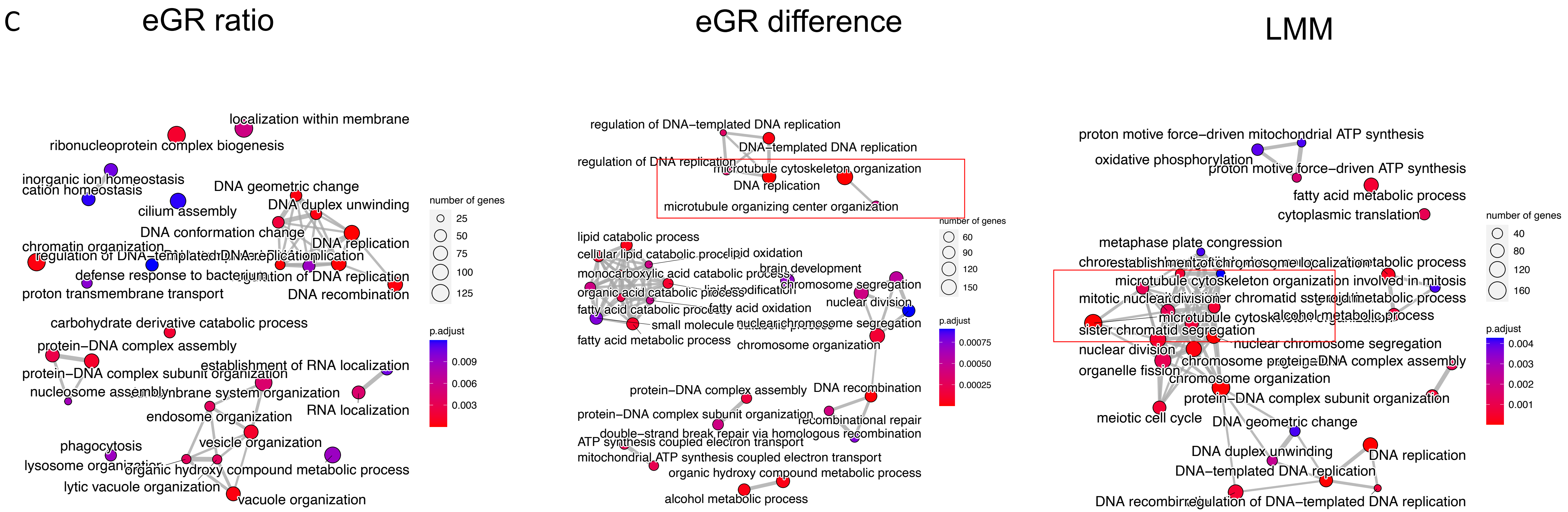
C
eGR ratio
localization within membrane
ribonucleoprotein complex biogenesis
inorganic ion homeostasis
cation homeostasis
cilium assembly
DNA geometric change
DNA duplex unwinding
DNA conformation change
DNA replication
chromatin organization
defense response to bacterium
proton transmembrane transport
DNA recombination
carbohydrate derivative catabolic process
protein−DNA complex assembly
protein−DNA complex subunit organization
establishment of RNA localization
nucleosome assembly
endosome organization
RNA localization
vesicle organization
phagocytosis
lysosome organization
organic hydroxy compound metabolic process
lytic vacuole organization
vacuole organization
number of genes
25
50
75
100
125
p.adjust
0.009
0.006
0.003
eGR difference
regulation of DNA−templated DNA replication
DNA−templated DNA replication
regulation of DNA replication
microtubule cytoskeleton organization
DNA replication
microtubule organizing center organization
lipid catabolic process
lipid oxidation
brain development
chromosome segregation
nuclear division
fatty acid oxidation
fatty acid metabolic process
chromosome organization
protein−DNA complex assembly
DNA recombination
protein−DNA complex subunit organization
recombinational repair
double−strand break repair via homologous recombination
ATP synthesis coupled electron transport
mitochondrial ATP synthesis coupled electron transport
organic hydroxy compound metabolic process
alcohol metabolic process
number of genes
60
90
120
150
p.adjust
0.00075
0.00050
0.00025
LMM
proton motive force−driven mitochondrial ATP synthesis
oxidative phosphorylation
proton motive force−driven ATP synthesis
fatty acid metabolic process
cytoplasmic translation
metaphase plate congression
microtubule cytoskeleton organization involved in mitosis
alcohol metabolic process
sister chromatid segregation
nuclear chromosome segregation
nuclear division
organelle fission
chromosome organization
protein−DNA complex subunit organization
meiotic cell cycle
DNA geometric change
DNA duplex unwinding
DNA replication
DNA−templated DNA replication
number of genes
40
80
120
160
p.adjust
0.004
0.003
0.002
0.001

Fig. S17D

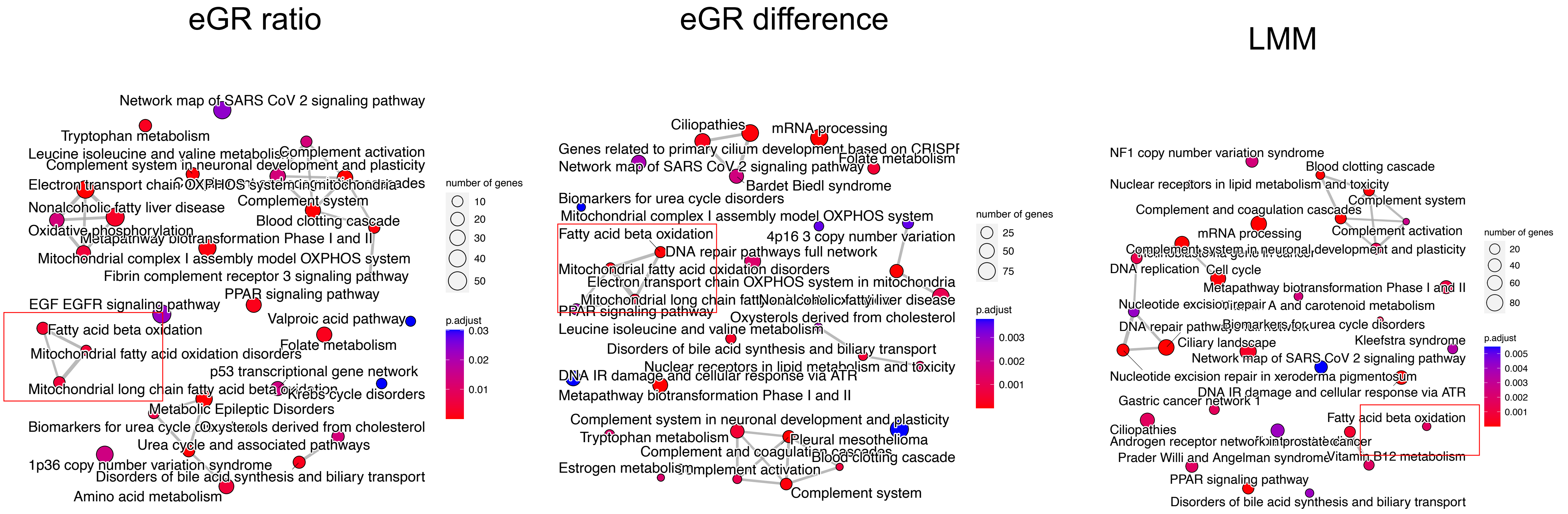
eGR ratio
Network map of SARS CoV 2 signaling pathway
Tryptophan metabolism
Leucine isoleucine and valine metabolism
Complement activation
Complement system in neuronal development and plasticity
Electron transport chain OXPHOS system in mitochondria
Nonalcoholic fatty liver disease
Complement system
Blood clotting cascade
Oxidative phosphorylation
Metapathway biotransformation Phase I and II
Mitochondrial complex I assembly model OXPHOS system
Fibrin complement receptor 3 signaling pathway
PPAR signaling pathway
EGF EGFR signaling pathway
Valproic acid pathway
Fatty acid beta oxidation
Mitochondrial fatty acid oxidation disorders
Folate metabolism
p53 transcriptional gene network
Mitochondrial long chain fatty acid beta oxidation
Krebs cycle disorders
Metabolic Epileptic Disorders
Biomarkers for urea cycle disorders
Oxysterols derived from cholesterol
Urea cycle and associated pathways
1p36 copy number variation syndrome
Disorders of bile acid synthesis and biliary transport
Amino acid metabolism
number of genes
10
20
30
40
50
p.adjust
0.03
0.02
0.01
eGR difference
Ciliopathies
mRNA processing
Genes related to primary cilium development based on CRISPR
Folate metabolism
Network map of SARS CoV 2 signaling pathway
Bardet Biedl syndrome
Biomarkers for urea cycle disorders
Mitochondrial complex I assembly model OXPHOS system
Fatty acid beta oxidation
4p16 3 copy number variation
DNA repair pathways full network
Mitochondrial fatty acid oxidation disorders
Electron transport chain OXPHOS system in mitochondria
Mitochondrial long chain fatty acid beta oxidation
Nonalcoholic fatty liver disease
PPAR signaling pathway
Oxysterols derived from cholesterol
Leucine isoleucine and valine metabolism
Disorders of bile acid synthesis and biliary transport
Nuclear receptors in lipid metabolism and toxicity
DNA IR damage and cellular response via ATR
Metapathway biotransformation Phase I and II
Complement system in neuronal development and plasticity
Tryptophan metabolism
Pleural mesothelioma
Complement and coagulation cascades
Blood clotting cascade
Estrogen metabolism
Complement activation
Complement system
number of genes
25
50
75
p.adjust
0.003
0.002
0.001
LMM
NF1 copy number variation syndrome
Blood clotting cascade
Nuclear receptors in lipid metabolism and toxicity
Complement system
Complement and coagulation cascades
mRNA processing
Complement activation
Complement system in neuronal development and plasticity
DNA replication
Cell cycle
Metapathway biotransformation Phase I and II
Nucleotide excision repair
Vitamin A and carotenoid metabolism
DNA repair pathways full network
Biomarkers for urea cycle disorders
Ciliary landscape
Kleefstra syndrome
Network map of SARS CoV 2 signaling pathway
Nucleotide excision repair in xeroderma pigmentosum
DNA IR damage and cellular response via ATR
Gastric cancer network 1
Fatty acid beta oxidation
Ciliopathies
Androgen receptor network in prostate cancer
Prader Willi and Angelman syndrome
Vitamin B12 metabolism
PPAR signaling pathway
Disorders of bile acid synthesis and biliary transport
number of genes
20
40
60
80
p.adjust
0.005
0.004
0.003
0.002
0.001

Fig. S17E

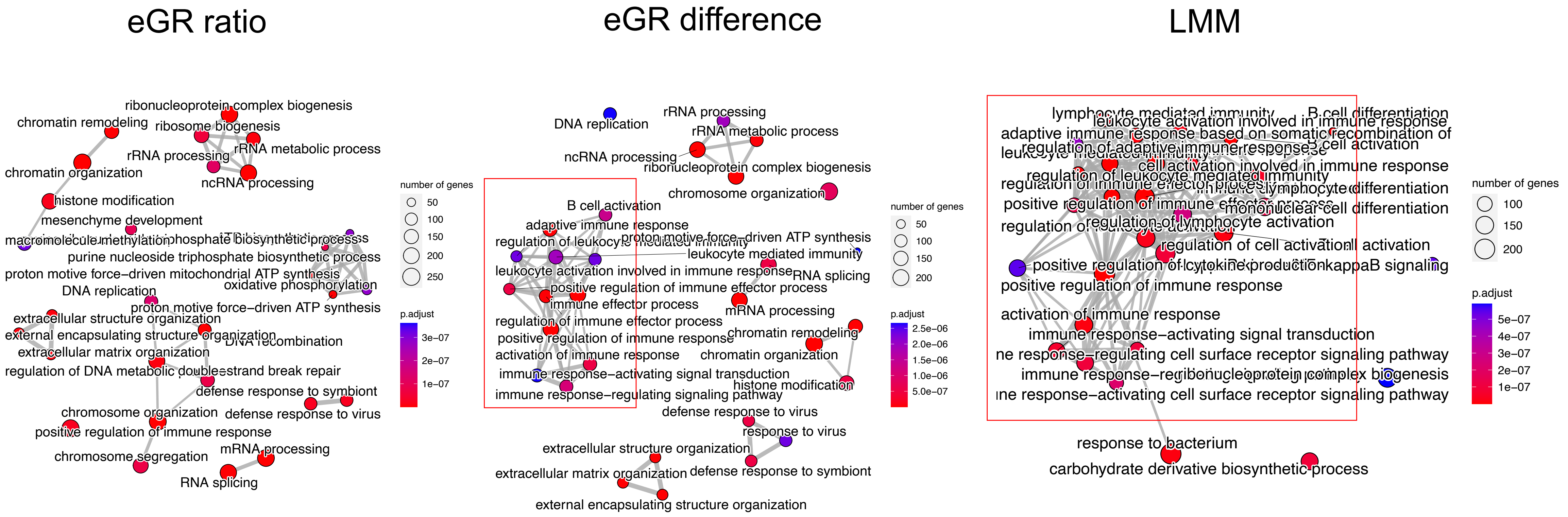

Supplementary figure 17. Enrichment map of GSEA. Edges connect overlapping gene sets. This way, mutually overlapping gene sets are tend to cluster together. The red rectangulated are drug-target related gene sets. A1) Cetuximab GO BP. A2) Cetuximab KEGG. B) Irinotecan GO BP. C) Paclitaxel GO BP. D) Sorafenib Wikipathway. E) PD-1 GO BP.
